# Engineering hydrophobic–aerophilic interfaces to boost N_2_ diffusion and reduction through functionalization of fluorine in second coordination spheres†

**DOI:** 10.1039/d3sc03002d

**Published:** 2023-08-01

**Authors:** Sakshi Bhardwaj, Sabuj Kanti Das, Ashmita Biswas, Samadhan Kapse, Ranjit Thapa, Ramendra Sundar Dey

**Affiliations:** a Institute of Nano Science and Technology (INST) Sector-81 Mohali 140306 Punjab India rsdey@inst.ac.in; b Department of Physics, SRM University Andhra Pradesh 522240 India

## Abstract

Ammonia is a crucial biochemical raw material for nitrogen containing fertilizers and a hydrogen energy carrier obtained from renewable energy sources. Electrocatalytic ammonia synthesis is a renewable and less-energy intensive way as compared to the conventional Haber–Bosch process. The electrochemical nitrogen reduction reaction (eNRR) is sluggish, primarily due to the deceleration by slow N_2_ diffusion, giving rise to competitive hydrogen evolution reaction (HER). Herein, we have engineered a catalyst to have hydrophobic and aerophilic nature *via* fluorinated copper phthalocyanine (F-CuPc) grafted with graphene to form a hybrid electrocatalyst, F-CuPc-G. The chemically functionalized fluorine moieties are present in the second coordination sphere, where it forms a three-phase interface. The hydrophobic layer of the catalyst fosters the diffusion of N_2_ molecules and the aerophilic characteristic helps N_2_ adsorption, which can effectively suppress the HER. The active metal center is present in the primary sphere available for the NRR with a viable amount of H^+^ to achieve a substantially high faradaic efficiency (FE) of 49.3% at −0.3 V *vs.* RHE. DFT calculations were performed to find out the rate determining step and to explore the full energy pathway. A DFT study indicates that the NRR process follows an alternating pathway, which was further supported by an *in situ* FTIR study by isolating the intermediates. This work provides insights into designing a catalyst with hydrophobic moieties in the second coordination sphere together with the aerophilic nature of the catalyst that helps to improve the overall FE of the NRR by eliminating the HER.

## Abstract 2

We engineered a catalyst *via* functionalization of fluorine in copper phthalocyanine grafted with graphene that forms a three-phase interface. This concept helps to suppress the parasitic HER process significantly with a high F.E. of NRR (49.3%).

## Graphical abstract

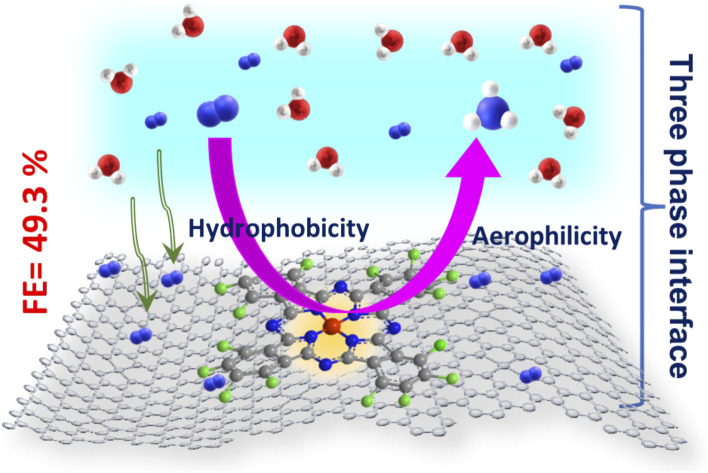

## Introduction

Ammonia has been realized as an essential commodity in the current world scenario where there is an utmost urge towards sustainability.^1^ At the moment the research community is engrossed in scaling up the electrochemical method of ammonia synthesis to get into the competition of large-scale ammonia production with the energy-intensive Haber–Bosch process.^2^ To date, a lot of progress has been witnessed in the eNRR in terms of catalyst development, method optimization and identification of false positive responses. However, the catalyst development genre is under extensive investigation and still has enough room for progress. Mainly, an electrocatalyst for the NRR is developed keeping in mind a few bottlenecks of the NRR like (a) selectivity towards N_2_ adsorption, (b) competitive hydrogen evolution reaction (HER),^3^ (c) catalysts having Lewis acid sites that influence N_2_ bond polarization, *etc.*^4^ Among them, the HER is looked down upon as it causes acute hindrance for a smooth NRR on the catalyst surface making industrial-scale ammonia synthesis a distant dream. This is because the NRR is a sluggish process involving a multiphase reaction pathway with six electron–proton transfer processes.^5^ At the same time, the HER needs only two protons and two electrons and is relatively fast.^6^ Moreover, in the aqueous medium, materials favour H adsorption due to unrushed N_2_ molecules which occupy most of the active sites that lead to parasitic hydrogen evolution by using up the majority of available electrons and reduces the selectivity for the NRR.^7^

Therefore, HER suppression will resolve the bottleneck problem of the NRR and will alleviate its selectivity and conversion efficiency for its practical application. Different strategies have been adopted to suppress the HER: (1) by regulating the accessibility of electrons and protons;^8^ (2) by modulating the chemical equilibrium of the HER; (3) by designing suitable catalysts. Out of these strategies, although the catalyst designing is an emerging field to mitigate the adsorption of H atoms, it has been less explored. In this respect, early transition metals (Ti, Zr, Y, and Sc)^9,10^ are in use due to their intrinsic HER restriction. Recently, atomically dispersed catalysts with late transition metals are widely explored for the NRR due to their unsaturated co-ordination configuration and maximum utilization of atoms.^3,11^ However, this metal specificity limits exploring wide-ranging materials for the NRR. As the NRR is highly sensitive to microenvironments, it is urgently needed to construct catalysts with rationally engineered active sites.^12^ So, it could be implemented for any metal/non-metal irrespective of its inherent properties and induce its reactivity towards the NRR, bypassing the otherwise feasible HER process. In this realm, for HER suppression and maximizing the mass transfer efficiency for the NRR, catalyst designing with induced hydrophobicity and aerophilicity on its surface is fascinating.^13,14^ Hydrophobicity introduces a triple-phase interface with a gas diffusion layer between the electrolyte and catalyst, which entraps N_2_ and provides more accessible region for the NRR. In the absence of the triple-phase interface, the catalyst surface is entirely in contact with the electrolyte making proton adsorption more susceptible and enhancing the HER.^15^ Despite HER suppression, the poor solubility of N_2_ in aqueous solution^16^ hinders the catalyst performance in the NRR.^17^ It means that N_2_ fixation will occur effectively in the case of fast diffusion of N_2_ towards the catalyst and the N_2_ bubbles formed on the catalyst surface should adhere purposely, showing an aerophilic effect.^18^ All in all, the catalyst should be designed with a hydrophobic and aerophilic nature for selective NRR. Aiming at this, tetrafluoroethylene (PTFE) or alkanethiols are used in some catalysts to improve hydrophobicity.^19,20^ This catalyst architecture accelerates the mass transport process of gas but the addition of an extra insulating layer reduces the electron transport and slows down the electrochemical process. Therefore, the challenge is to improve gas diffusion without hampering the active catalytic sites. More importantly, besides this catalyst improvisation, it is crucial to realize the actual mechanism ongoing in these types of materials such that it could be further vitalized towards a more efficient NRR.

In general, most of the electrocatalytic processes take place in the transition metal centre, if present, where the metal–ligand moiety is present in the first coordination sphere (CS). Although the first CS is directly involved in the catalytic process, the role of the second CS is sometimes found to be exorbitant. Second coordination spheres, usually stabilize by H-bonding, π–π interactions, chemical bonding and even the electrostatic effect of the neighbouring atoms/molecules, can alter the reaction pathway in many ways. This leads to enhanced reaction kinetics *via* facilitating the diffusion of reactant molecules to the catalyst surface or the adsorption of the intermediates during the reaction's progress. In recent years, there have been a few reports, where the catalysts are designed based on the presence of second CSs to enhance the O_2_, N_2_ and CO_2_ reduction reactions.^21–23^ Unfortunately, most of the literature has not shown the detailed reaction pathway on how the protonation takes place after they got adsorbed on the catalyst surface, which is a major gap in previous studies. It is therefore indispensable to design an electrocatalyst for the NRR, where the second CS would help N_2_ diffusion and it gets adsorbed in the first CS, where the water concentration is reasonably better and sufficient for protonation during its reduction.

Therefore, to address this long-standing issue, for the first time, we have introduced the role of primary and secondary spheres in N_2_ adsorption and its subsequent reduction for the synthesis of ammonia. Herein, we have synthesized fluorinated copper phthalocyanine grafted on exfoliated graphene (F-CuPc-G) as a catalyst, which, as expected, is found to be highly hydrophobic and aerophilic in nature in aqueous electrolytes. The catalyst is engineered in such a way that fluorine moieties are available in the second CS (hydrophobic layer) and the active metal center is in the first CS. Therefore, there occurs fast N_2_ diffusion due to the formation of a triple-phase interface in the second CS and also active sites in the first CS available for the adsorption of N_2_ molecules and their subsequent reduction, leading to an efficient eNRR. Along with its hydrophobic nature, the catalyst is found to be aerophilic, which adheres gaseous N_2_ molecules on the catalyst surface and slows down the HER process, which is almost unavoidable in aqueous electrolytes and promotes the ammonia conversion.^8^ Moreover, a theoretical study demonstrates how hybridizing F-CuPc with graphene provides better electronic conductivity by altering the electronic structure of active sites and more density of states (DOS) near the Fermi level, facilitating better electronic transport from active sites to the electrode surface. Thus, this work highlights the importance of the co-existence of hydrophobic and aerophilic nature in the catalyst that assists in suppressing the HER and accelerating the NRR with high faradaic efficiency.

## Results and discussion

**Fig. 1:**
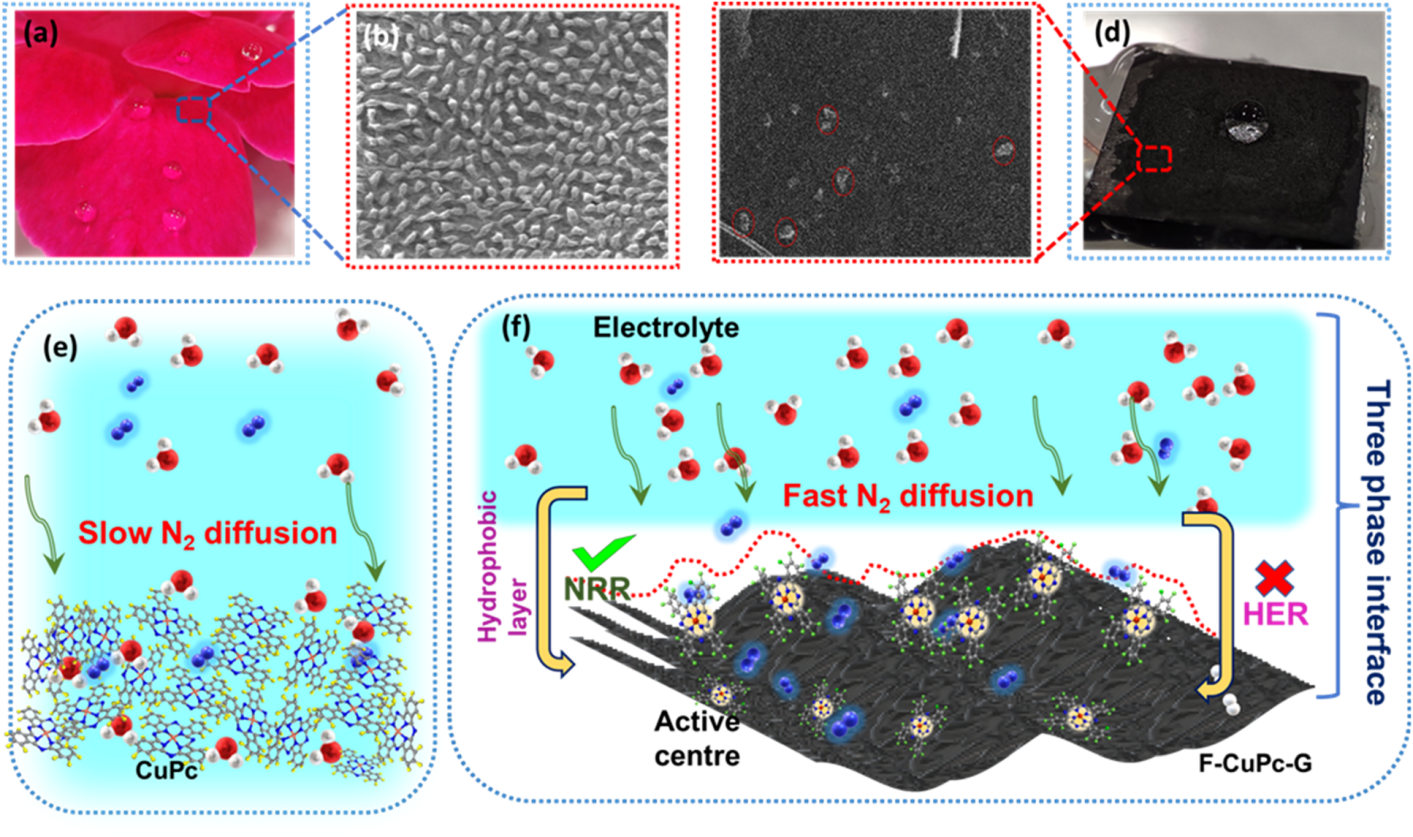
(a) Optical image of rose petals with water droplets, FE-SEM image of (b) a rose petal, (c) F-CuPc-G, (d) optical image of F-CuPc-G with an electrolyte droplet, (e) scheme representing the slow diffusion of N_2_ in the electrolyte in the case of CuPc, and (f) fast diffusion of N_2_ in the electrolyte in the case of F-CuPc-G. The yellow, grey, blue, red, green and pink spheres represent the hydrogen, carbon, nitrogen, oxygen, fluorine and copper atoms.

**Fig. 2:**
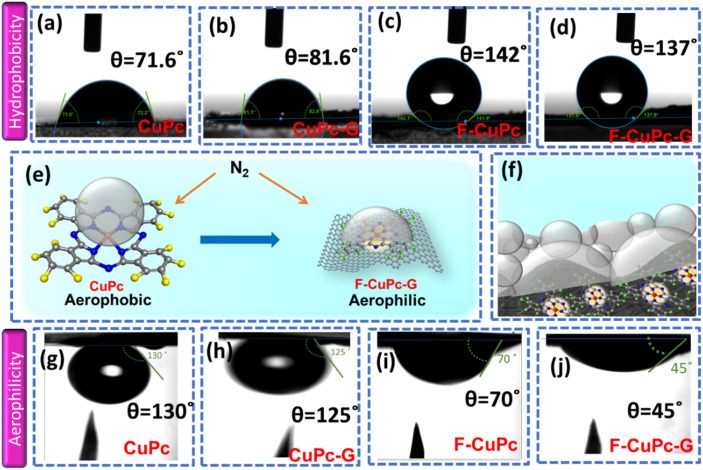
The contact angles of electrolyte bubbles measured on the surface of (a) CuPc, (b) CuPc-G, (c) F-CuPc, and (d) F-CuPC-G; (e) scheme illustrating the aerophobic behaviour of CuPc and aerophilic behaviour of F-CuPc-G, (f) overall scheme showing the three phase interface of F-CuPc-G presenting hydrophobicity as well as aerophilicity, and the contact angles of N_2_ bubbles measured on the surface of (g) CuPc, (h) CuPc-G, (i) F-CuPc, and (j) F-CuPc-G.

The wettability of rough surfaces in nature remains an exciting subject for exploration and inspiration of many innovations. Surface roughness in combination with the chemistry of materials determines the wettability of the surface.^24^ For the conversion of a gaseous molecule in aqueous medium at the solid catalyst surface, the concept of hydrophobicity plays a crucial role in terms of gas entrapment at the solid–liquid–gas (three-phase) interface. Thus, getting inspiration from biological surfaces like rose petals (Fig. 1a) having nanostructures as seen in their field emission scanning electron microscopy (FESEM) image (Fig. 1b) responsible for their hydrophobicity, we added some functional groups to the existing catalyst (copper phthalocyanine: CuPc) specifically to improve its hydrophobicity and for swift N_2_ diffusion and entrapment on the catalyst active site. Here, we synthesized fluorinated copper phthalocyanine (F-CuPc) using a reported method^25^ as explained in the Experimental section and grafted it on electrophoretically exfoliated graphene as an underlying conductive support. The catalyst F-CuPc-G displayed a structural resemblance to that of rose petals as shown in FESEM (Fig. 1c). Hence, as expected, the catalyst was highly hydrophobic (Fig. 1d), as can be seen from the persistent water droplet over the catalyst surface similar to that on the rose petals (Fig. 1a). During the eNRR, the CuPc catalyst was all over flooded with electrolytes, as shown in Fig. 1e and hence, there occurred slow diffusion of N_2_ and was more prone to the HER. On the other hand, the as-synthesized catalyst (F-CuPc-G) manifested a better way for N_2_ diffusion owing to the hydrophobic layer, dominant at the three-phase interface (Fig. 1f) which was attributed to the presence of fluorine edge-functionalities that constitute the secondary sphere of the catalyst F-CuPc-G. Once the N_2_ molecules swept through the secondary sphere, their adsorption over the primary coordination sphere, followed by the subsequent protonation, got reinforced due to the decent availability of protons in the local hydrophilic environment of the first CS. This not only helped to mitigate the competitive HER over the catalyst active site successfully but also provided a route to justify the source of protons required for the protonation steps of the NRR despite the hydrophobic nature of the catalyst. The extent of hydrophobicity in the primary material as well as the control samples was analyzed by contact angle measurement. Fig. 2a–d show that with fluorination and graphitization the hydrophilic behaviour of CuPc turned to hydrophobic in F-CuPc-G as the contact angle changed from 71.6° to 137°.^26^ The high surface roughness and low surface energy attained due to the presence of graphene and fluorine, respectively, were synergistically responsible for the hydrophobicity of F-CuPc-G.^20^ Moreover, these properties imparted the characteristic of water adhesion to F-CuPc-G, similar to rose petal adhesion (Fig. S1†). The adhesive ability of the material keeps the water in contact, which is helpful for the protonation step in the NRR process. Our hydrophobic and adhesive catalyst (F-CuPc-G) possessed another significant feature, that is the aerophilic property, which presented a strong N_2_ adsorption layer and interaction of the nitrogen bubbles with the catalyst as compared to CuPc as seen in Fig. 2e. The hydrophobic catalyst with aerophilic nature provided rapid N_2_ transport and kept it trapped even under the electrolyte flooding state (Fig. 2f).^27^ It was revealed that nitrogen bubbles were in the “pinning” state^28^*i.e.*, aerophobic on CuPc and CuPc-G (Fig. 2g and h), while its angle decreased on the surface of F-CuPc and F-CuPc-G showing that the aerophilicity induced N_2_ gas layer on the catalyst slowed down the HER process on the active site, due to less availability of H^+^ ions (Fig. 2i and j).

**Fig. 3:**
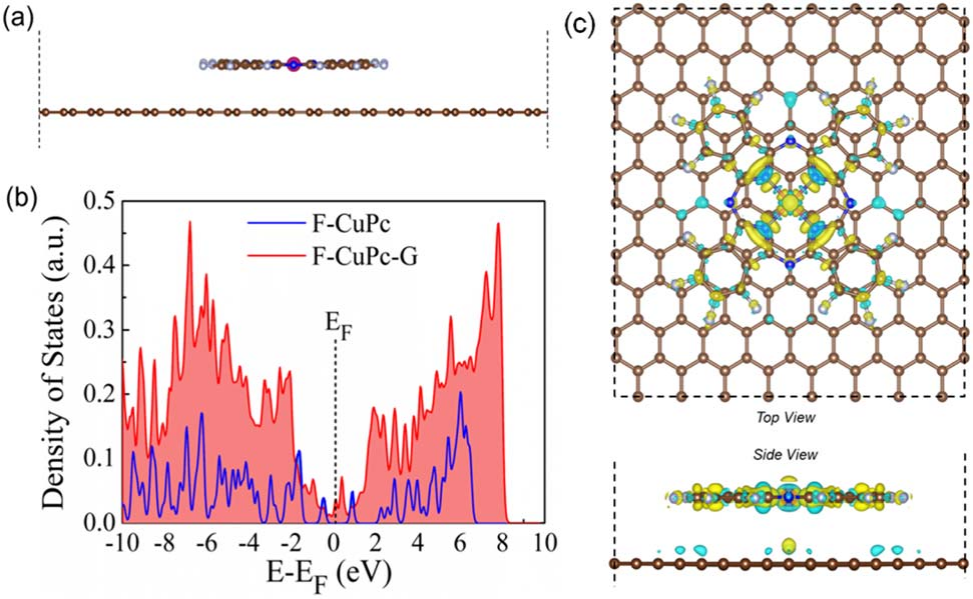
(a) Optimised model structure of the F-CuPc-G heterostructure. (b) Plot of the density of states for the F-CuPc and F-CuPc-G structures, and (c) the charge density difference plots for the F-CuPc-G system. Yellow and blue lobes indicate electron accumulation and depletion layers, respectively (isosurface value = 0.00032 e Å^−3^).

The synthesized F-CuPc material was confirmed by Fourier-transform infrared spectroscopy (FTIR) and Raman analysis (Fig. S2 and S3†).^29^ The FTIR spectrum of F-CuPc shows peaks at 603, 765, 965, and 1152 cm-1 due to hexa-decafluoro substituents, respectively, which are absent in the FTIR spectrum of CuPc, as shown in Fig. S2,† indicating successful functionalization of fluorine in the CuPc matrix. However, owing to its very low conductivity in the order of 1–8 MΩ, F-CuPc receded in the NRR performance as discussed in the later sections. Therefore, to improve its conductivity F-CuPc was intercalated with graphene nanosheets using single-step electrophoretic exfoliation of graphite plates in an acidic medium to form a F-CuPc-graphene π-conjugated system as shown in Fig. S4† with three different contents (catalysts F-CuPc-G_0.01_, F-CuPc-G_0.02_ (F-CuPc-G), and F-CuPc-G_0.03_) and as expected, the conductivity of the hybrid F-CuPc-G catalyst improved (Fig. S5†). The loading of F-CuPc into the graphene was measured by inductively coupled plasma mass spectroscopy (ICP-MS) analysis. From the calculation we found the presence of 120.96, 147 and 181.44 ppb of Cu in the catalysts F-CuPc-G_0.01_, F-CuPc-G_0.02_ (F-CuPc-G), and F-CuPc-G_0.03_ respectively in 0.07 mg catalyst used in the experiment (Table S1†). Theoretical calculations were further carried out to determine the density of states (DOS) for the heterostructure of F-CuPc with graphene to understand the effect of underneath graphene (Fig. 3a and b). It was observed that a larger number of states occurred near the Fermi level for F-CuPc-G as compared to pristine F-CuPc, which was the reason for the higher conductivity of the F-CuPc-G system that was responsible for the better electron transfer from the catalyst to the electrode during the eNRR, whereas pristine graphene did not have the states at the Fermi level.^30^ Through charge density difference analysis, we visualized the charge re-distribution over F-CuPc molecules due to the underneath graphene layer (Fig. 3c).

**Fig. 4:**
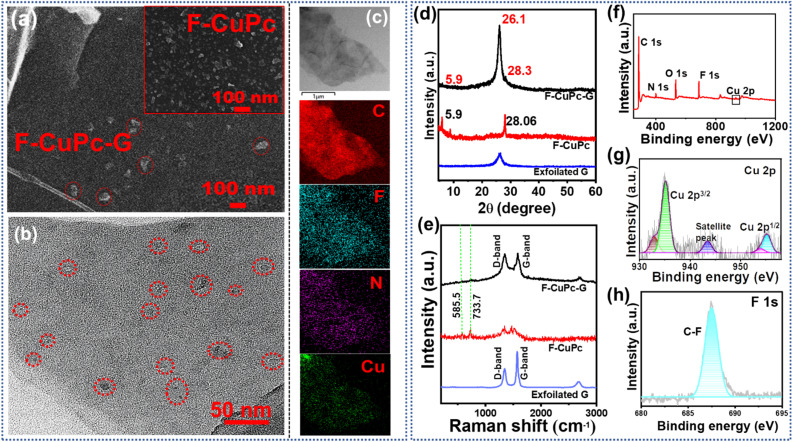
(a) FE-SEM image of F-CuPc-G; inset: F-CuPc, (b) TEM image of F-CuPc-G; (c) TEM elemental mapping of F-CuPc-G, shows the distribution of C, F, N and Cu; (d) XRD pattern and (e) Raman spectra of F-CuPc-G, F-CuPc and exfoliated graphene. (f) The full survey XPS spectra of F-CuPc-G; high resolution XPS spectra of (g) Cu 2p, and (h) F 1s in F-CuPc-G.

The FE-SEM image in Fig. S6† presented the exfoliated graphene sheets. The granular structures of F-CuPc as shown in inset of Fig. 4a were also found to be dispersed on the intercalated graphene sheets depicting the presence of F-CuPc throughout the sheet (Fig. 4a). The fine graphene sheets with F-CuPc moieties stacked between them could be observed in the transmission electron microscopy (TEM) image (Fig. S7a and b†). Fig. 4b distinctly shows the encircled F-CuPc dispersed over the graphene sheets. Furthermore, the images of elemental-mapping of carbon, copper, fluorine and nitrogen were displayed vividly, presenting their distribution (Fig. 4c).

The X-ray diffraction (XRD) analysis (Fig. 4d) showed the characteristic peak at 2*θ* = 26.1° denoting the (002) plane representing the sp^2^ hybridization of graphitic carbon-like exfoliated graphene.^31^ The XRD peaks of F-CuPc reflected well in the XRD pattern of F-CuPc-G and the little shift in the peaks may be due to the π–π interactions between F-CuPc and graphene sheets. In the Raman spectra of F-CuPc-G, two distinct peaks were obtained at 1347.4 cm^−1^ and 1577.2 cm^−1^ for D and G-bands reflecting the degree of graphitization in the material as in exfoliated graphene^32^ (Fig. 4e). The peaks found in F-CuPc overlaid some peaks in F-CuPc-G depicting the good incorporation of F-CuPc on the graphene sheets.^33^ Furthermore, to get more information about the surface properties of F-CuPc-G, an X-ray photoelectron spectroscopy (XPS) study was conducted.^34^ The XPS survey spectrum of F-CuPc-G showed distinct peaks for C, N, O, F and Cu confirming their presence in the hybrid material (Fig. 4f). The high-resolution XPS spectra of Cu (Fig. 4g) depicted peaks centered at 935.2, 955.1 and 943.5 eV for Cu 2p_3/2_, Cu 2p_1/2_ and Cu satellite peaks respectively.^35^ The F 1s peak at 688 eV represents the C–F bonds (Fig. 4h). The deconvoluted C 1s and N 1s spectra are shown in Fig. S8 and S9.†^36^ All these characterisation methods confirm the successful incorporation of F-CuPc into the 2D layered graphene sheets to form F-CuPc-G. The N_2_ adsorption/desorption study is crucial to the catalytic efficiency of a porous catalyst. To analyse the surface area and pore size distribution (PSD) of the samples (Fig. S10a–c†), the isotherms were obtained where the hybrid material possessed *a* surface area of 210 m^2^ g^−1^ with a PSD of 3.8 nm. Besides this, the exfoliated graphene and F-CuPc complex have a surface area of 38 m^2^ g^−1^ and 3 m^2^ g^−1^ along with a PSD of 1.5 nm, 3.8 nm and 2.7 nm, 7.4 nm, respectively. In the isotherm of F-CuPc, a very sharp increase of N_2_ adsorption/desorption in the higher pressure region was observed, indicating the presence of interparticle porosity (mesoporous) of the complex. In the isotherm of exfoliated graphene both micro- and meso-pores are present. The electrochemical exfoliation technique in the presence of pi electron containing F-CuPc facilitated the exfoliation of graphene sheets through pi–pi interaction. Due to incorporating the F-CuPc complex into the pi–pi stacked layered graphene material, the pore size distribution region shifted towards the mesoporous region and the surface area was also increased. The mesoporous hybrid structure increased the accessibility of the N_2_ containing electrolyte towards the catalytically active centre of the catalyst and consequently increased the catalytic efficiency. Also, the fast kinetics between the electrode and electrolyte was possible due to low internal resistance in F-CuPc-G as suggested by the Nyquist plot (Fig. S11†). The electrochemical surface area (ECSA) was also found to be maximum for F-CuPc-G as it had the highest value of double layer capacitance (*C*_dl_) as shown in Fig. S12.†

**Fig. 5:**
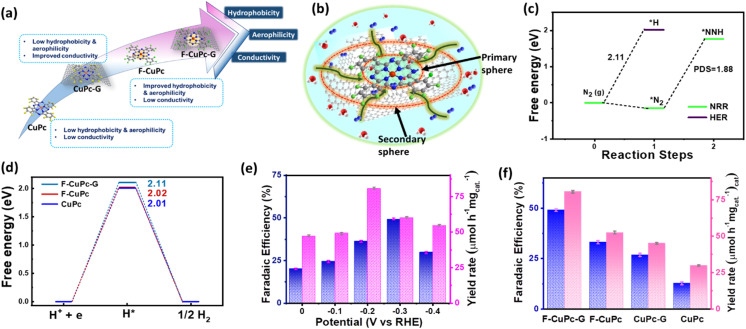
(a) Scheme representing the limitations and improvements of different catalysts, (b) scheme showing the mechanistic pathway of N_2_ diffusion through primary and secondary spheres in F-CuPc-G, (c) free energy profile of N_2_ and NNH adsorption on F-CuPc-G, (d) free energy profile of the HER for F-CuPc-G, F-CuPc and CuPc systems. The bar plot diagrams illustrating the FE and yield rate (e) at different potentials for F-CuPc-G, and (d) for different catalysts.

The eNRR is mainly governed by a few parameters that primarily include nitrogen adsorption followed by its bond polarizability and protonation steps to yield NH_3_. The conductivity of an electrocatalyst and sufficiency of d-electrons enable a better bond polarizability of N_2_ by the facile electron donation and π-back donation strategy.^37^ This was the reason why we chose CuPc with the Cu active site coordinated with four N atoms and graphitized the material by means of electrophoretic exfoliation to obtain CuPc-G. Nevertheless, the main difficulty in aqueous electrolytes is that, although the proton sufficiency enables a better N_2_ protonation, there remains a predominant probability of proton adsorption over N_2_ adsorption. This, in turn, retards the yield rate and faradaic efficiency of NH_3_ synthesis. It is challenging to simultaneously play with both adsorption and protonation parameters to elevate the overall reaction kinetics. This was the primary focus of this work to enhance the N_2_ sufficiency around the active center without any detrimental effect on the subsequent NRR steps, which was successfully brought about by incorporating fluorine as a part of the active catalyst. The edge fluorine atoms induced local hydrophobicity and aerophilicity at the three-phase interface that made way for easy N_2_ diffusion across the secondary coordination sphere of the catalyst. Emphatically, the aerophilicity of the material leads to the formation of an N_2_ gas diffusion layer over the catalyst, which bypassed the tendency of water molecules to make their way to the catalyst before proton adsorption. So, here the role of the primary coordination sphere came into play, wherein the local hydrophilic environment provided protons to the Cu-adsorbed-N_2_ molecules to proceed further the protonation steps as schematically elaborated in Fig. 5a and b. The Gibbs free energy calculations of F-CuPc-G revealed a negative free energy and hence spontaneous N_2_ adsorption on the active site while disfavouring the high positive energy (2.11 eV) driven proton adsorption. In fact, the first protonation step (*NN to *NNH), which is the potential determining step for the NRR, involved 1.88 eV, better than the proton adsorption on the active site (Fig. 5c). This free energy value for the HER was highest for F-CuPc-G compared to CuPc and F-CuPc inherently restricting the HER (Fig. 5d). Thus, our catalyst designing played a dual role of promoting N_2_ adsorption and its first protonation despite the locally available protons in the primary coordination sphere of the catalyst F-CuPc-G.

This approach is expected to have a broader impact on the FE of ammonia production by the electrocatalyst during the NRR. In order to verify it, the electrochemical process was carried out in a three-electrode system in a 1.0 M Na_2_SO_4_ electrolyte. Before the electrochemical studies, the N_2_ gas was trapped in 0.5 M H_2_SO_4_ and 0.1 M KOH to free it from any kind of gaseous impurity. The trapped gas was hence checked with the gas chromatography technique, as shown in Fig. S13† and then purged into the electrolyte for the experiments. The yielded NH_3_ in each experiment was quantified by using a UV-vis spectrophotometer by the indophenol blue method using the calibration curve obtained with a known concentration of NH_3_ (Fig. S14 and S15†). This was also done for acid and base trap solutions for the respective adventitious NH_3_ and NO_*x*_ impurities to check the purity of N_2_ feed gas (Fig. S16–S21†). For both the trap solutions, no detectable absorbance was obtained under the UV-visible spectrophotometer (Fig. S22 and S23†). The side product hydrazine (N_2_H_4_) was detected by the Watt and Chrisp method^38^ (Fig. S24 and S25†). The fast N_2_ diffusion by fluorination of CuPc and improvement in the conductivity by graphitization make F-CuPc-G a contender for an efficient NRR that can be seen from change in the current densities in linear sweep voltammetry (LSV) in Ar and N_2_ saturated atmospheres (Fig. S26†). Over the potential range, 0.0 to −0.4 V *vs.* RHE chronoamperometry (CA) was run for an hour at each potential as shown in Fig. S27.† The corresponding aliquot electrolyte was incubated for 2 hours for NH_3_ and 15 minutes for N_2_H_4_ detection using their respective coloring agents as explained in the ESI.†^39^ Then, UV-vis spectra were taken for NH_3_ and N_2_H_4_ at different potentials (Fig. S28 and S29†). The bar plot (Fig. 5e) presented the maximum NH_3_ yield of 80.58 μmol h^−1^ mg_cat._^−1^ at −0.2 V and highest FE (49.3%) at −0.3 V *vs.* RHE using eqn (2) and (3) and the data have been compiled in Table S2.† As expected, this FE value indicated a successful suppression of the HER (Fig. S30†) at the active site, which is an essential requirement for improving the NRR kinetics in ambient aqueous electrolytes. There was no trace of any peak in the UV-visible spectra at 460 nm corresponding to the side product (N_2_H_4_) formation as determined by the Watt and Chrisp method,^38^ which forecasted the material selectivity towards NH_3_ synthesis over the entire potential range. Such high ammonia production could be attributed to the fast mass transfer of N_2_ due to the induced hydrophobic–aerophilic nature of the catalyst. To further confirm the role of F and underlying graphene support, various control samples such as CuPc, CuPc-G and F-CuPc were investigated (Fig. S31†) for one-hour CA tests. Fig. 5f clearly reveals that F-CuPc-G wins the league with the highest NH_3_ yield and FE (Fig. S32 and Table S3†) and also, the comparison with the literature is given in Table S4.† F-CuPc acts as a n-type semiconductor^40^ that has low intrinsic conductivity. Therefore, the graphitization of F-CuPc improved the proton coupled electron transfer for nitrogen fixation that was anyway reflected in the electrocatalytic performance. Also, the amount of F-CuPc in graphene solution was varied (0.01 mmol, 0.02 mmol and 0.03 mmol) to optimize its electrocatalytic NRR response and the optimized F-CuPc-G (0.02 mmol) showed the highest FE and yield (Fig. S33 and S34†).

**Fig. 6:**
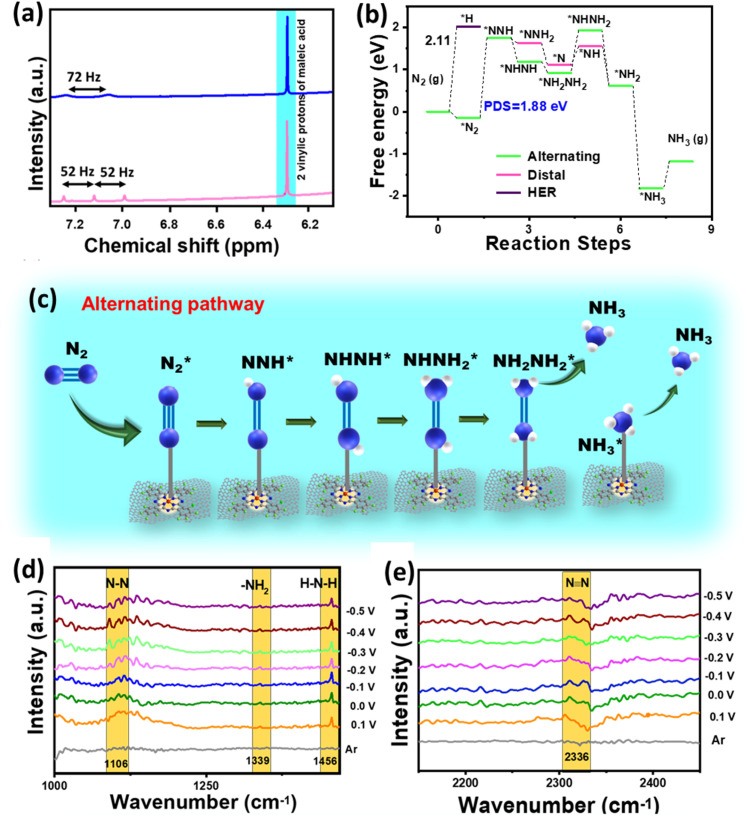
(a) Isotope labelling experiment displaying ^1^H NMR spectra obtained after the NRR in 1 M Na_2_SO_4_ with ^14^N_2_ (pink) and ^15^N_2_ (blue) as feed gases, (b) Free energy profile of the NRR for F-CuPc-G, (c) scheme showing the reaction mechanism of F-CuPc-G during NRR follows alternating pathway. (d and e) *In situ* ATR-FTIR spectra obtained for F-CuPc-G.

Besides this, various blank CA experiments for F-CuPc-G with Ar and N_2_ saturation and at open circuit potential (OCP) in 1 M Na_2_SO_4_ were performed to check that the NH_3_ produced during the experiment was only from the feed N_2_ gas and not from any other nitrogenous impurity. Almost no NH_3_ was detected in either case, as can be seen from the spectra (Fig. S35†) and the bar plot (Fig. S36†). However, the active material F-CuPc-G contained N atoms coordinated to the metal active center. This adds to the benefit of the doubt whether the material N contributes to the NH_3_ so formed. This necessitates the verification of ammonia production using an isotope labeling experiment. In this, the distinguishable signals with different coupling constants were obtained in ^1^H-NMR for ^14^NH_4_^+^ and ^15^NH_4_^+^. Here, the triplet with 52 Hz and the doublet with 72 Hz were attributed to ^14^NH_4_^+^ and ^15^NH_4_^+^, respectively in ^1^H-NMR (Fig. 6a). Furthermore, quantitative analysis was done using maleic acid using eqn (4) in the ESI.† The FE calculated from this method was almost similar in both cases of ^14^N_2_ and ^15^N_2_ as the feed gas.

Besides noteworthy NRR activity, it is very important that the catalyst be stable and maintain hydrophobic–aerophilic nature even after long run in the aqueous electrolyte. As fluorine functionalization of CuPc provides a hydrophobic–aerophilic interface, it is essential to check the elemental composition and contact angle after the stability test. It was found from the CA run that the catalyst was stable up to 16 h (Fig. S37†) with almost no change in the curve and as expected almost the same NH_3_ yield rate was obtained (inset of Fig. S37†). The elemental composition and structural conformation after the stability experiment were examined by XRD, Raman, XPS and elemental composition analysis (Fig. S38 and Table S5†). All the characterization methods showed almost no change or insignificant change in the results obtained after the stability test compared to their respective data obtained before the stability test. This proved the robustness of our catalyst even after prolonged NRR activity. The hydrophobic–aerophilic interface was checked *via* contact angle measurements after a long-term stability test. It was found that the contact angle in the case of hydrophobicity changes from 137° to 132° (Fig. S39†) and from 45° to 40° (Fig. S39†) in the case of aerophilicity, illustrating persistent hydrophobic–aerophilic behaviour of the catalyst. This further proved the swift movement of N_2_ molecules by restricting the protons and the consistent N_2_ adsorption ability of the catalyst throughout the prolonged experimental hours. In addition, F-CuPc-G exhibited very good cyclability over five cycles of CA for one hour each under the same conditions (Fig. S40†). We obtained almost similar UV-spectra for all (Fig. S41†) and consequently, the NH_3_ yield rate and FE were repetitively reproducible (Fig. S42†).

A theoretical study was performed to understand the effect of F ends and the origin of higher NRR activity of F-CuPc based systems. In this context, two model structures of CuPc with H ends and F-CuPc with F ends (Fig. S43a and b†) were considered. To find the thermodynamic stability of F-CuPc, we performed molecular dynamics simulations at various temperatures (300 K, 500 K, 700 K, and 900 K).^41,42^ Here, we did not observe any bond breaking or distortion of the structure (Fig. S44a–d†). However, we observed only a small change in the planarity of the F-CuPc molecule. Therefore, we confirmed the high thermodynamic stability of F-CuPc. Furthermore, DFT calculations were performed to elucidate the reaction mechanism of the NRR. The free energy profile of N_2_ and NNH indicates a small increment in the adsorption energies in F-CuPc as compared to CuPc (as shown in Fig. S45a†). So, we plotted the full free energy profile of the NRR for F-CuPc (Fig. S45b†) and F-CuPc-G (Fig. 6b) that gives the step of *N_2_ to *NNH as a potential determining step (PDS).^43^ It was found to be minimum for F-CuPc-G. Following theoretical studies^44^ from the literature and the free energy pathway (Fig. 6b), F-CuPc-G was found to obey an alternating pathway with the least Gibbs free energy. Therefore, Fig. 6c presents the full alternating pathway adopted on the catalyst, F-CuPc-G. It is more preferred over the distal pathway due to the higher adsorption energy of *NHNH than the *NNH_2_ intermediate.^45^ The synergetic roles of fluorine ends and the underneath graphene layer are influential in fast N_2_ diffusion and reducing the competitive hydrogen evolution reaction on the Cu site. Therefore, it is confirmed that F-CuPc-G is highly selective for nitrogen adsorption as compared to hydrogen, which leads to improve NRR activity over the HER. Thus, this work provided a strong impression that the hydrophobic–aerophilic catalyst is highly recommendable for gaseous electrocatalytic reactions.

To better understand the reaction intermediates, an *in situ* attenuated total reflectance-Fourier transform infrared (ATR-FTIR) spectroscopy study of F-CuPc-G during the eNRR was carried out. As shown in Fig. 6d, the interfacial FTIR spectrum in an argon medium does not show any characteristic peak. However, in a N_2_ saturated atmosphere, at different potentials from 0.1 to −0.5 V *vs.* RHE, after running chronoamperometry for 600 s, three peaks located at 1106, 1339 and 1456 cm^−1^ were obtained, which are assigned to N–N stretching, –NH_2_ wagging and H–N–H bending, respectively.^46^ The peak at 2326 cm^−1^ appeared due to the adsorption of the nitrogen molecule as N≡N got polarised (Fig. 6e). Additionally, there was no peak for N_2_H_4_ observed, which is consistent with the UV-vis spectroscopy results.

## Experimental section

### Synthesis

Tetrafluorophthalonitrile and CuCl_2_ were purchased from Sigma Aldrich and were used as received. Potassium hydroxide (KOH) (analytical grade) obtained from Sigma Aldrich, hydrochloric acid (HCl), sulphuric acid (H_2_SO_4_) and potassium sulphate (Na_2_SO_4_) were purchased from Merck chemicals. All other reagents used in this study were of pure analytical grade and were used without any further purification. Graphite plates were purchased from Merck chemicals. All aqueous solution was prepared using Millipore water.

0.2 g of 1 mmol of tetrafluorophthalonitrile and 0.135 g of 1 mmol CuCl_2_ were added and heated at 200 °C for 3 h, giving rise to a dark blue/green solid. After it was allowed to cool down to an ambient temperature, it was vacuum filtrated and washed with water and acetone, respectively. The acetone filtrate was evaporated and F-CuPc was obtained.

The as-synthesised F-CuPc was further graphitized by a facile graphene exfoliation method. The experimental setup for this graphene synthesis is very simple equipped with two electrodes (graphite plates), an electrolyte and a DC source. Two graphite plates (1 × 1 cm^2^) having identical dimensions (one acts as a cathode and other as an anode) were dipped in an acidic solution containing 0.5 M H_2_SO_4_ and different amounts of F-CuPc (0.1 mmol, 0.2 mmol and 0.3 mmol named F-CuPc-G_0.01,_ F-CuPc-G_0.02,_ and F-CuPc-G_0.03_) at a static potential of 2.5 V for 30 minutes. There occurred exfoliation of graphene plate after a few minutes. The loose black flakes split up from the plate which would lead to a dark suspension of graphene flakes peeled off from the graphite plate and this plate started vanishing gradually. This black solution was then subjected to ultra-sonication for 30 to 120 minutes to get well dispersed graphene solution containing F-CuPc.

## Conclusions

In summary, here we present the concept of hydrophobic–aerophilic behaviour of a F-CuP-G catalyst and the significance of the primary and secondary coordination spheres associated with it. This approach not only favoured N_2_ gas diffusion and adsorption across the three-phase interface but also clarified the source of protons required to carry forward the successive protonation steps despite the hydrophobic layer over the catalyst surface. This is a significant achievement of this work, as the hydrogenation step of NRR on the hydrophobic materials reported in the literature so far was unexplored. The catalyst significantly suppressed the parasitic HER process with a high NH_3_ yield rate of 80.53 μmol h^−1^ mg_cat._^−1^ at −0.2 V and exceptionally high FE (49.3%) at −0.3 V *vs.* RHE. DFT and *in situ* FTIR studies were carried out to determine the reaction intermediates and unravel the energetically favoured associative alternating reaction pathway for the NRR on the catalyst, F-CuPc-G. This study thus opens up a new domain of research to explore the field of electrochemical NH_3_ synthesis with a strategy of fast N_2_ diffusion and HER suppression.

## Data availability

All the experimental data and computational details related to this work are available either in the manuscript or in the ESI.†

## Author contributions

S. Bhardwaj, S. K. Das and R. S. Dey designed the work. S. K. Das synthesized the catalyst. S. Bhardwaj has done all the material charaterizations and electrochemical test. S. Bhardwaj has written the manuscript. S. K. Das and A, Biswas have assisted S. Bhardwaj in writing and mechanism study. S. Kapse and R. Thapa have carried out all the theoretical calculation and corresponding writing part. R. S. Dey supervised the work and corrected the manuscript. All the authors have checked the final version of the manuscript and approve it for submission.

## Conflicts of interest

There are no conflicts to declare.

## Supplementary Material

SC-014-D3SC03002D-s001
