# Supplementary material for: Engineering hydrophobic–aerophilic interfaces to boost N2 diffusion and reduction through functionalization of fluorine in second coordination spheres
Source: Chem Sci. 2023 Aug 1;14(33):8936–45. doi: 10.1039/d3sc03002d (PMC10445478; doi:10.1039/d3sc03002d)
Supplement: SC-014-D3SC03002D-s001 [file SC-014-D3SC03002D-s001.pdf]

Electronic Supplementary Material (ESI) for Chemical Science.
This journal is © The Royal Society of Chemistry 2023

Electronic supplementary information (ESI)

Engineering hydrophobic-aerophilic interface to boost N_2 diffusion and reduction through functionalization of fluorine in second coordination sphere

Sakshi Bhardwaj[†a], Sabuj Kanti Das[†a], Ashmita Biswas[a], Samadhan Kapse[b], Ranjit Thapa[b] and Ramendra Sundar Dey*[a]

[a] Energy Environmental Unit (EEU)
Institute of Nano Science and Technology
Sector – 81, Mohali-140306, Punjab, India.
E-mail: rsdey@inst.ac.in
† Contributed equally.

[b] Samadhan Kapse, Ranjit Thapa
Department of Physics
SRM University AP, Andhra Pradesh

Table of contents

1. Instrumentation
2. Electrochemical measurements and detection techniques
3. Computational details
4. Supporting Figures and Tables
5. References

1. Instrumentation:

X-ray diffraction (XRD) patterns of the samples were obtained using X-ray diffractometer (Bruker D8 Advances instrument) with Cu-Kα (λ = 1.5406 Å) radiation with an acceleration voltage of 40 KV in the 2θ range from 10° to 70°.

The detailed surface microstructure of samples was analyzed by field emission scanning electron microscope (FESEM) (JEOL JSM-7600F).

The Raman spectra of the samples was obtained using WITEC Focus Innovations Alpha-300 Raman confocal microscope under an excitation laser of 532 nm and Fourier transform Infrared spectroscopy (FT-IR) was carried out on an Agilent technology Cary 600 series FTIR instrument at room temperature.

X-ray photoelectron spectroscopy (XPS) spectrometer (K-Alpha 1063) instruments in an ultrahigh vacuum chamber ($7X10^{-9}$ torr) using Al-K_{α} radiation (1486.6 eV) was used to investigate the surface elemental composition and bonding configuration of the prepared samples.

^{1}H spectra were measured with a 400 MHz Bruker Avance II 400 NMR spectrometer. Chemical shifts are reported in parts per million (δ) calibrated by using tetramethylsilane as an internal standard for samples in [D6] DMSO.

2. Electrochemical measurements and detection techniques

Electrochemical characterizations were performed using CHI 760E electrochemical workstation that included linear sweep voltammetry (LSV) and chronoamperometry. The NRR was carried out in H-cell with three electrode system under ambient conditions and the two compartments of H-cell were separated by Nafion-115 membrane. The membrane is cleaned by boiling in ultrapure water for 1 h and then treated with H_2O_2 (5%) aqueous solution for another 1 h at 80 °C. After that it was dipped in 0.5 M H_2SO_4 at 80 °C for 3 h and at last in water for 6 h. The working electrode used was graphite plate with area 1*1 cm^2, Ag/AgCl as reference electrode and Pt as counter electrode. The potential in RHE can be related as:

$$E_{RHE} = E_{Ag/AgCl} + 0.0591\, pH + 0.210 \qquad eq. 1$$

1.0 M Na_2SO_4 is used as the working electrolyte which is fed with Ar and N_2 for 30 minutes prior to the experiment.

N_2 gas purification: For the removal of possible contaminants in the $^{14}N_2$ gas, commercially purchased from Sigma Aldrich was passed firstly through acid (0.05 M H_2SO_4) and then, alkaline (0.1 M KOH) traps as it is expected that the adventitious NH_3 will get trapped into the acid trap and the base trap would capture NO_x impurities. A detailed analysis of the NH_3 and NO_x impurities in the gas were checked with UV-vis spectroscopic methods. Before being purged into the electrocatalytic cell, the feed gas was collected and checked with Gas Chromatography to detect the NO or N_2O contamination in the purified gas.

NO_x determination: NO_x contamination was checked by using the N-(-1-naphthyl)- ethylenediamine dihydrochloride spectrophotometric method. The chromogenic agent was obtained by dissolving sulfanilic acid (0.5 g) in deionized water (90 ml) and acetic acid (5 ml), followed by adding N-(1-naphthyl)-ethylenediamine dihydrochloride (5 mg) and bringing the solution to 100 ml. The prepared solution was covered and protected from light. The chromogenic agent (1 mL) was mixed with 4 mL of the investigating solutions. After standing in darkness for another 15 min, the absorption spectrum was measured using an ultraviolet-visible spectrophotometer at 546 nm.

NH_3 detection:
Indophenol blue method
From cathodic reaction chamber, 5 mL of the aliquot was taken and added 20 µL of phenol (1mg mL^{-1}) followed by 12.5 µL of sodium nitroferricyanide ($C_5FeN_6Na_2O$) in water. Then, adjust the pH of solution approx. 10 using NaOH and trisodium citrate solution and then added 100 µL NaOCl to it. The solution mixture was incubated for 1 h and then UV-vis analysis is done to detect the ammonia evolved during the reaction. The concentration of NH_3 formed was determined by the calibration curve obtained from a set of solutions containing known concentration of NH_4Cl in 1 M Na_2SO_4. To these solutions, the above-mentioned reagents were added and incubated for 1h to check the absorbance. The fitted plot presented a good linearity of absorbance with concentration of NH_3 which is given by;
y = 0.4566x + 0.0137; R^2 = 0.995

Hydrazine (N_2H_4) detection:

Watt and Chrisp method
The coloring agent for detection of N_2H_4 was prepared by dissolving 0.2 g of para-(dimethylamino) benzaldehyde in 10 mL of ethanol and 1 mL concentrated HCl. To the 2 mL of aliquot, 2 mL of the prepared coloring agent was added and then so formed mixture was incubated in dark for 15 minutes before performing the UV-vis characterization. The fitted plot presented a good linearity of absorbance with concentration of NH_3 which is given by;
y = 0.50786x + 0.165; R^2 = 0.987
FE % and Yield rate:
FE for NRR is defined as the ratio of amount of electric charge used for NH_3 production to the total charge passed through the electrodes during the process of electrolysis. By colorimetric method the amount of NH_3 produced is determined. FE can be calculated as follows:

$$FE = \frac{3F \times c_{NH_3} \times V}{17 \times Q} \qquad eq. 2$$

where 3 is the no. of electrons needed to produce one molecule of NH_3, F is Faraday's constant, c_{NH_3} is concentration of NH_3 produced, V is the volume of the electrolyte used and Q is the total charge passed through the electrodes.
The NH_3 yield rate (R_{NH_3}) is given by:

$$R_{NH_3} = \frac{c_{NH_3} \times V}{m \times t} \qquad eq. 3$$

where t is the time of reaction and m is the catalyst mass.
$^{15}N_2$- isotope labeling Experiment:
$^{15}N_2$ (Sigma Aldrich, 98 atom% $^{15}N_2$) was used as the feeding gas in the isotope labelling experiment. Before feeding the electrolyte solution with $^{15}N_2$, the electrolyte was degassed using Ar for an hour. After electrolysis the electrolyte was taken out and concentrated, followed by addition of 0.01 M maleic acid and 0.4 ml of D6-DMSO.

Quantification of ammonia concentration from NMR:

The catholyte solution was concentrated to 1 mL and 400 µL was taken out of it for NMR analysis. This was subsequently added with 50 µL of 0.01 M maleic acid solution followed by DMSO-d6 and subjected to ^{1}H-NMR study. The obtained peaks were integrated and by using the following eq. 4, the concentration of NH_3 was quantified and matched with that obtained from UV–visible spectroscopic method.

$$\frac{I_{sample}}{I_{standard}} = \frac{H_{sample} * C_{sample}}{H_{standard} * C_{standard}} \qquad eq. 4$$

where *I* stands for the integral values, H stands for the number of protons (4 in case of sample NH_4^+ and 2 in case of the vinylic protons of maleic acid) and *C* stands for the concentrations of the sample and standard (0.01 M for maleic acid).

3. Computational details

Theoretical calculations are performed using density functional theory with plane-wave technique implemented in the Vienna Ab initio Simulation Package (VASP)[1]. The core electrons and electron exchange-correlation interaction are described through Projected Augmented Wave (PAW) pseudopotentials[2] and Generalized Gradient Approximation (GGA) proposed by Perdew, Burke and Ernzerhof[3] respectively. In the plane-wave basis set, we used the optimised value of cutoff energy of 450 eV that is obtained through convergence test. The energy convergence criteria are set to be 10^{-5} eV and 10^{-4} eV for electronic self-consistent loop and ionic relaxation loop respectively. Brillouin zone sampling within monkhorst pack scheme is obtained using the gamma point for CuPc as well as F-CuPc and 5x5x1 K-point grid for F-CuPc-G system. The interaction between repeating images is neglected using the vacuum of 20 Å in respective directions. Free Energy Profile is the effective analysis to investigate the reaction mechanism and catalytic activity of materials towards nitrogen reduction reaction (NRR). In this, Gibbs free energies (G) of all reaction steps are estimated by using equation, G = E+ZPE–TS-neU[4], where E is DFT energy, ZPE and TS are the zero-point energy and entropic term respectively. n is the number of electrons in step and U is the applied potential at the electrode. The ZPE and TS terms for the adsorbed intermediates are small and negligible. whereas DFT energies, TS terms and ZPE standard values for the free molecules are given below, which we calculated to plot the free energy profile of NRR.

**Molecule**	**E (eV)**	**TS (eV)**	**ZPE (eV)**	**G (eV)**
**H_2**	-6.77	0.41	0.27	-6.91
**NH_3**	-19.41	0.58	0.92	-19.07
**N_2**	-16.19	0.59	0.15	-16.63

4. Supporting Figures and Tables

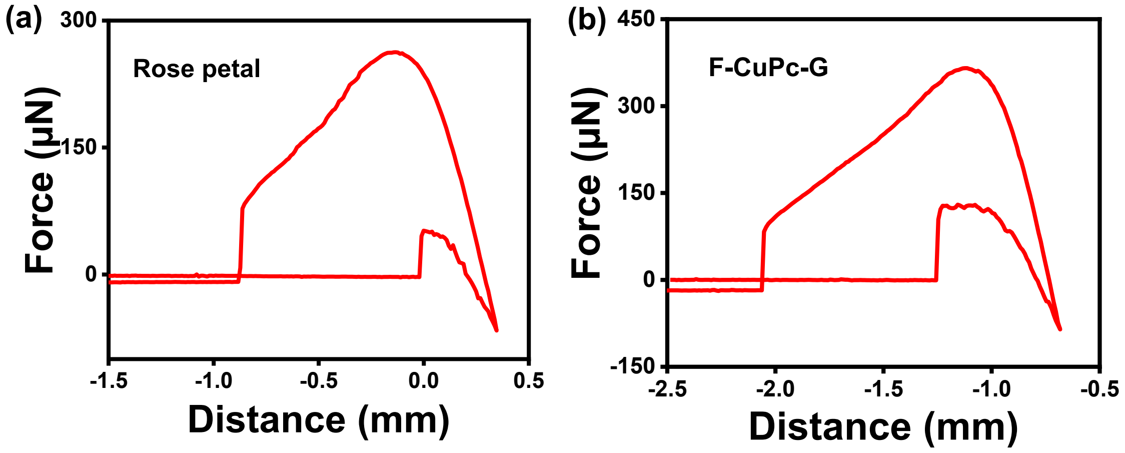

Figure S1. Adhesion force measurements of (a) Rose petal, (b) F-CuPc-G.

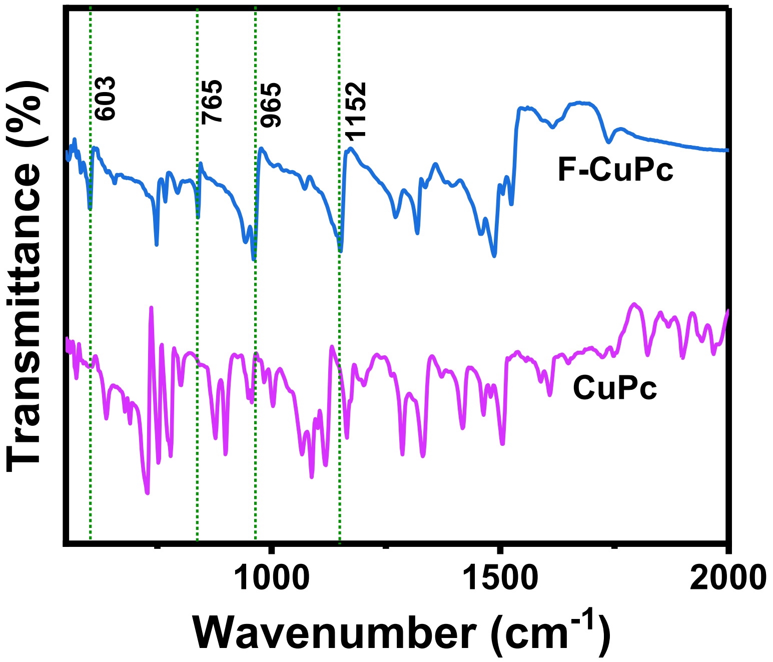

Figure S2. FTIR spectra in transmission mode of F-CuPc.

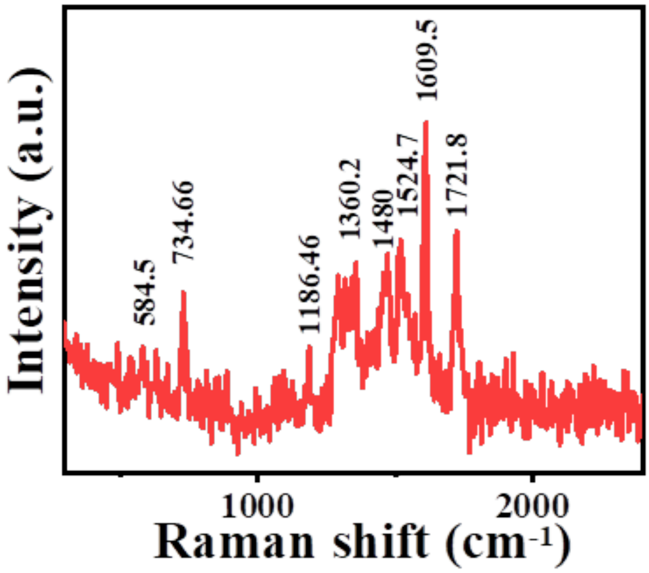

Figure S3. Raman spectra of F-CuPc.

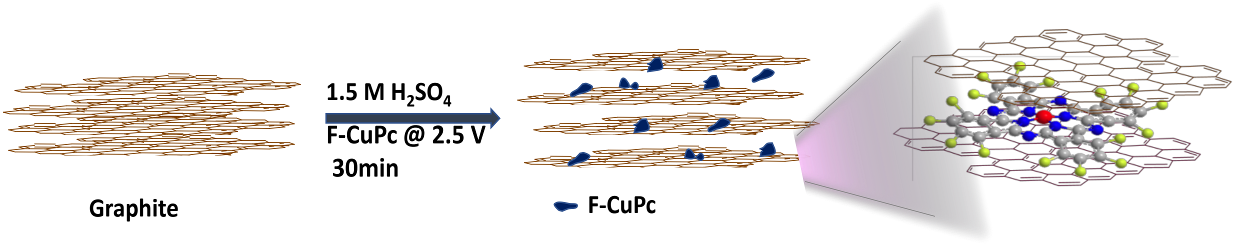

Figure S4. Exfoliation of graphite plates in the presence of F-CuPc to synthesise F-CuPc-G

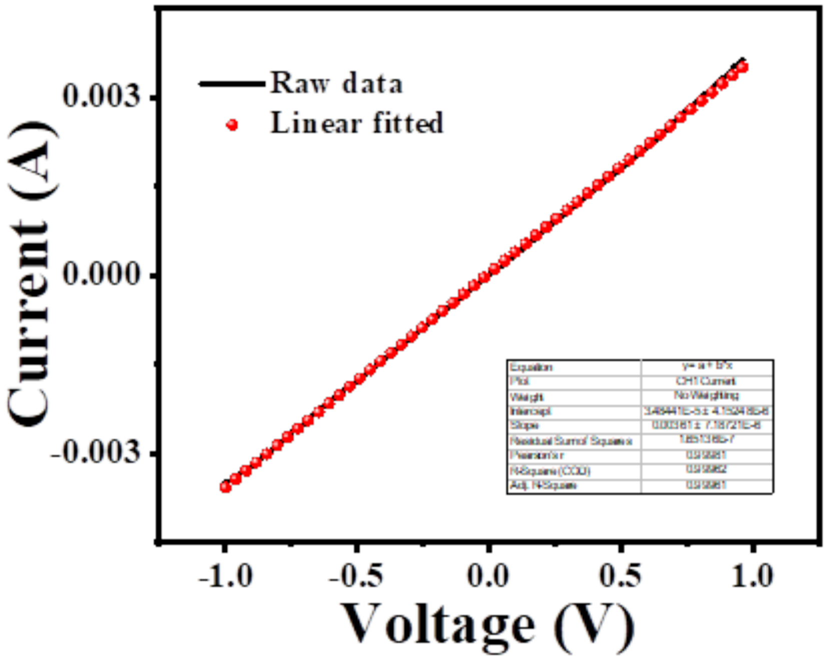

Figure S5. Conductivity measurement of F-CuPc-G.

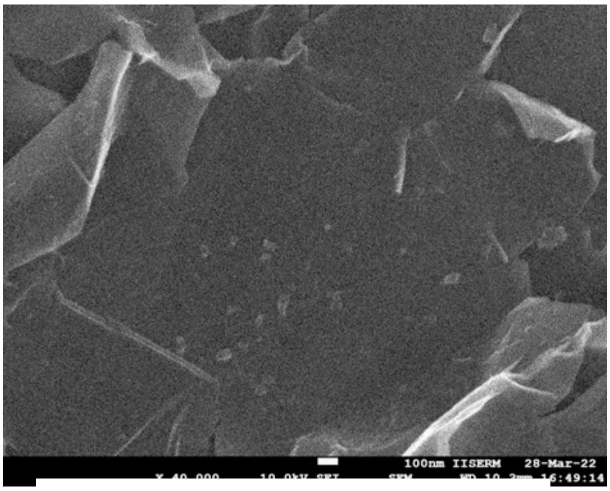

Figure S6. FESEM image of F-CuPc-G.

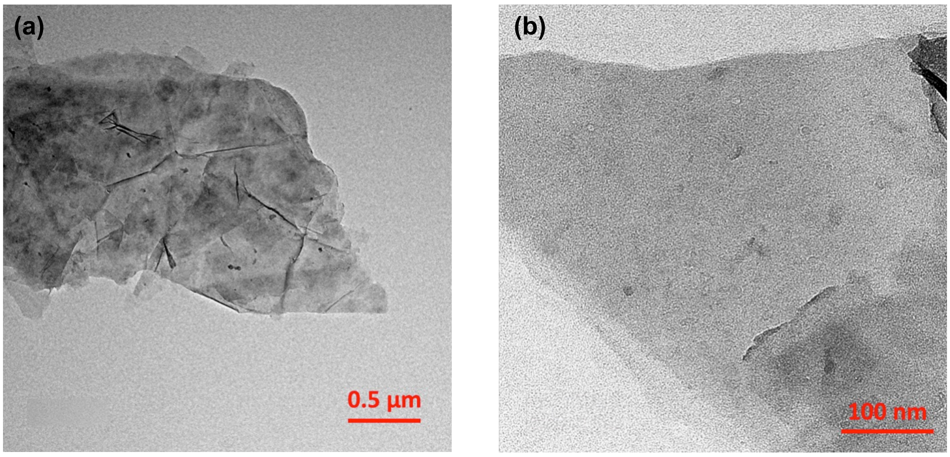

Figure S7 (a,b). TEM images of F-CuPc-G.

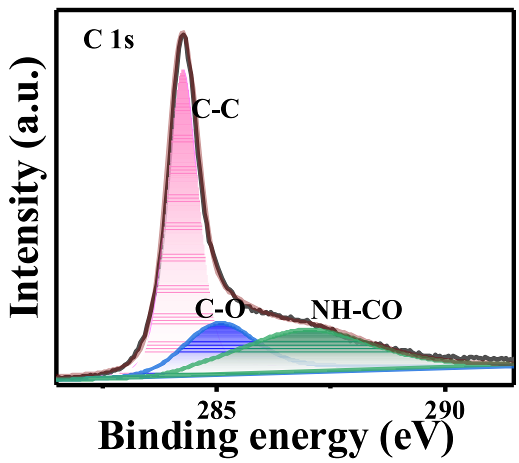

Figure S8. High resolution XPS spectra of C1s in F-CuPc-G

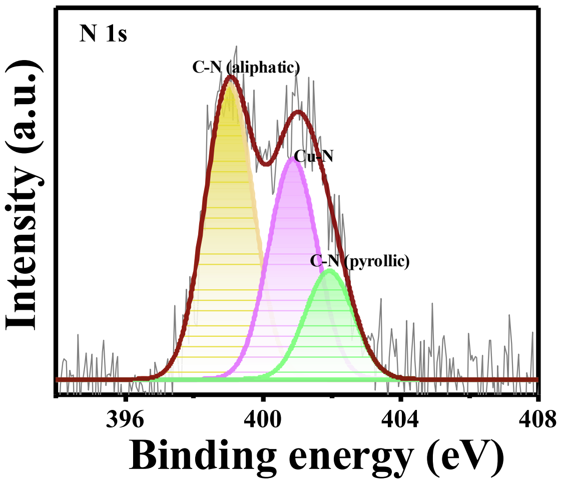

Figure S9. High resolution XPS spectra of N 1s in F-CuPc-G.

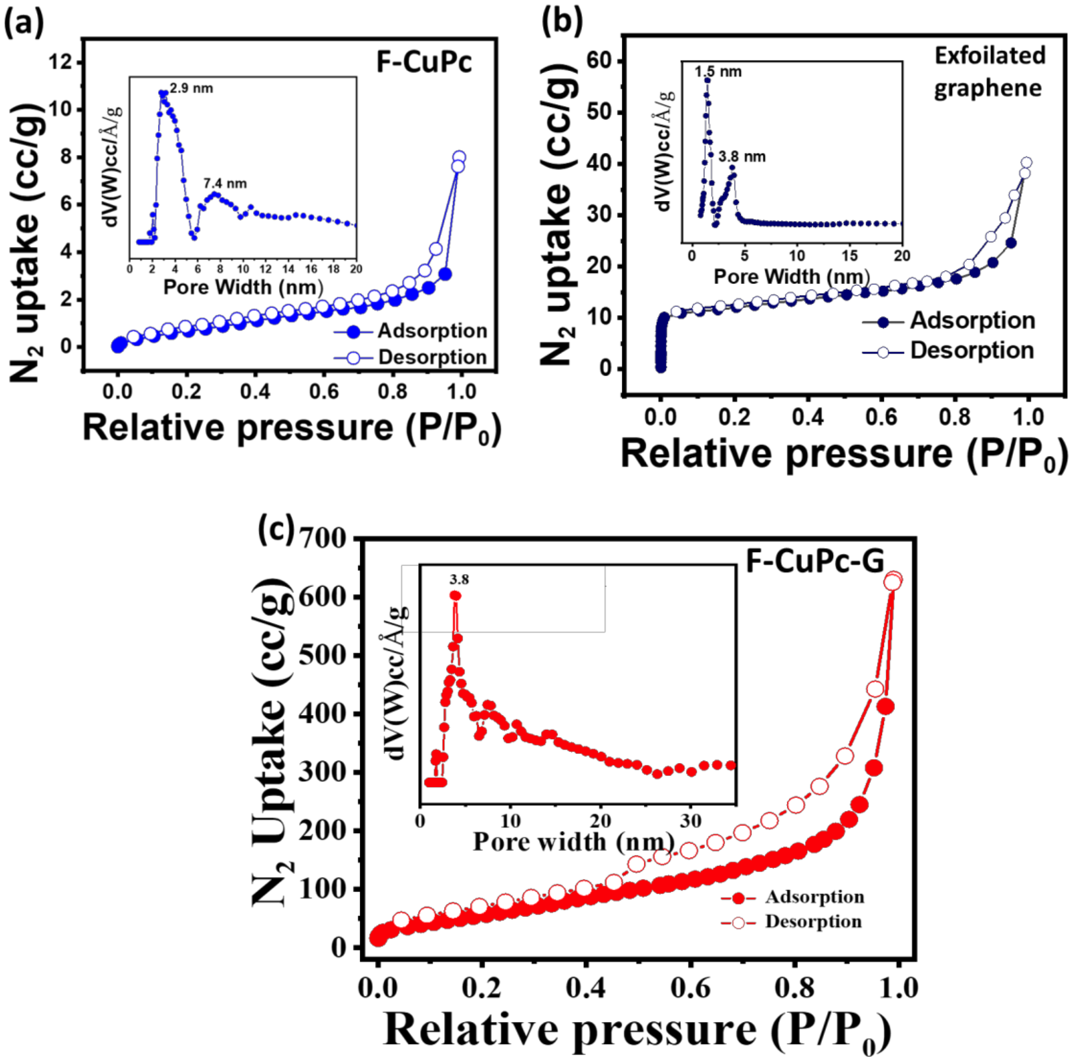

Figure S10. N_2 adsorption-desorption isotherms and inset: the corresponding pore size distribution curves of (a) F-CuPc (b) exfoliated graphene (c) F-CuPc-G.

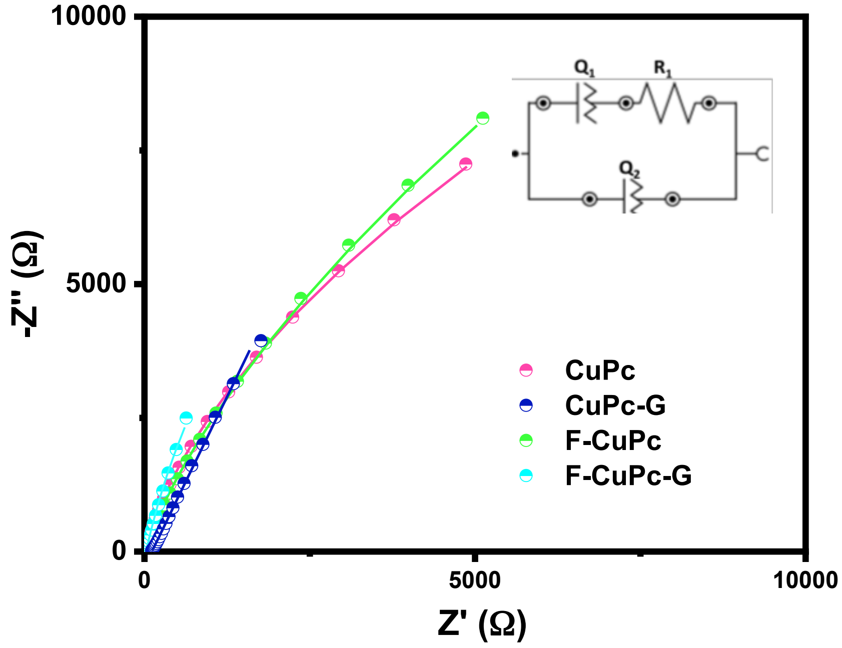

Figure S11. Nyquist plots of CuPc, CuPc-G, F-CuPc and F-CuPc-G, the equivalent circuit used to fit the EIS data.

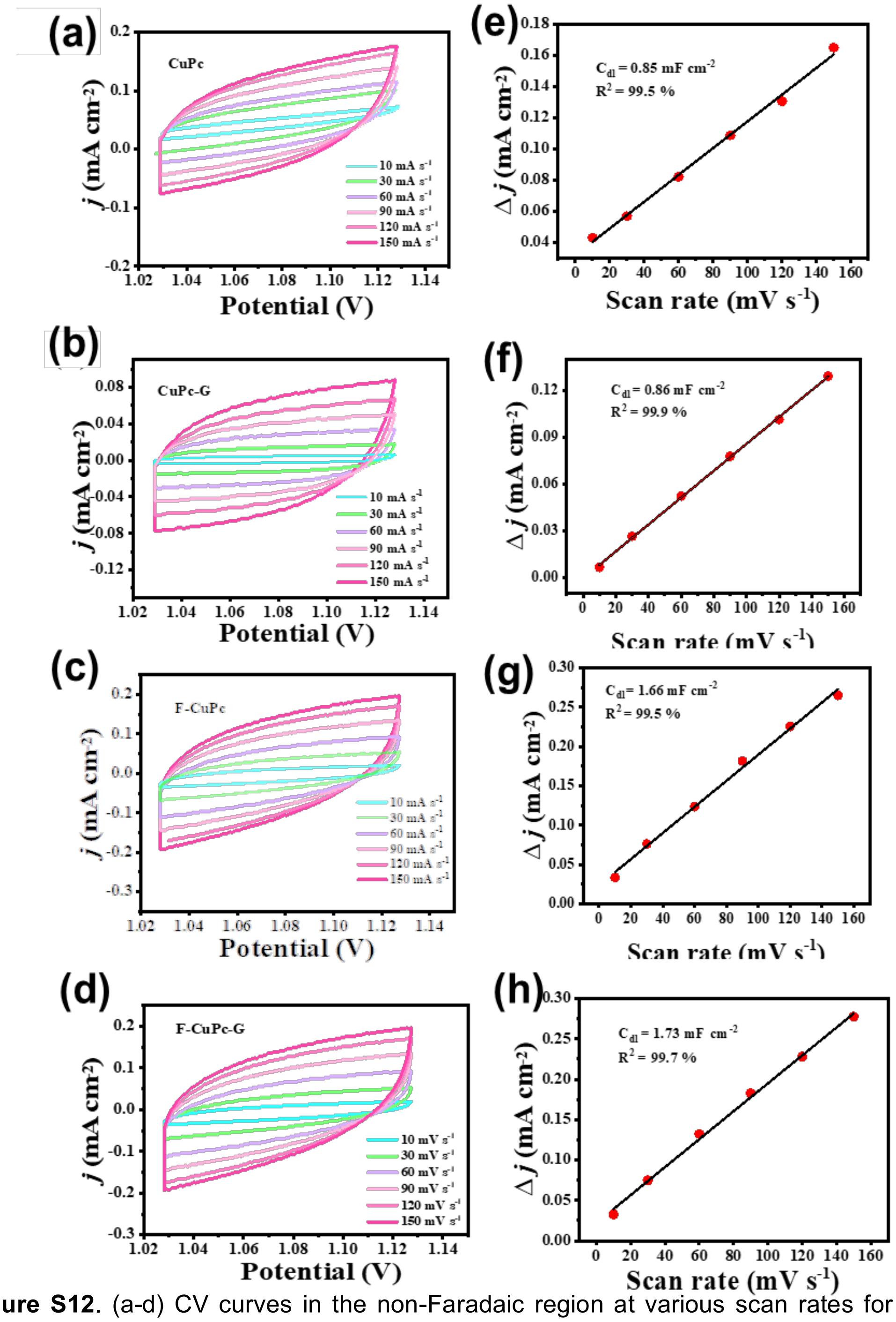

Figure S12. (a-d) CV curves in the non-Faradaic region at various scan rates for all the synthesized catalysts and (e-h) Linear fits of difference of anodic and cathodic current densities extracted from the CV curves for all the synthesized catalysts with respect to different scan rates at definite potentials, where the slope represents twice the double layer capacitance (C_{dl}).

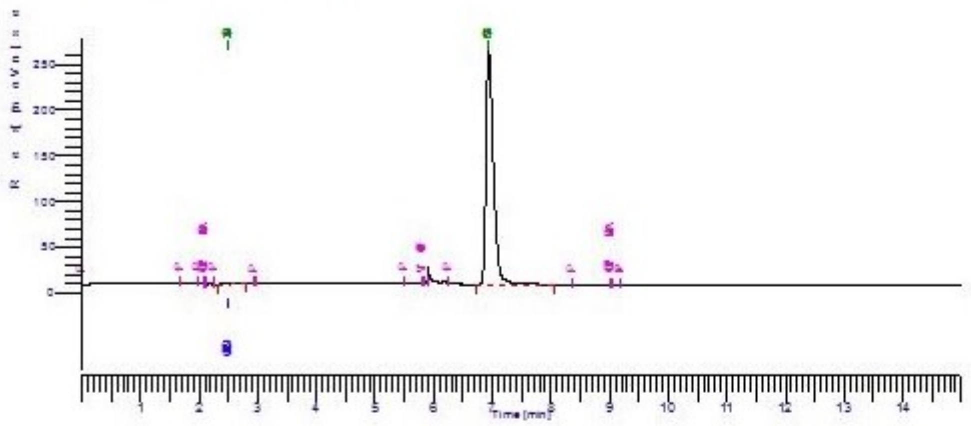

INST Mohali

Peak #	Component Name	Time [min]	Area [uV*sec]	Height [uV]	Area [%]	Norm. Area [%]	Raw Amount	Concentration ppm
1	carbon dioxide	2.496	16565.99	1791.36	0.73	0.73	23.3258	23.3258
2		6.935	2261041.67	259177.20	99.27	99.27	2.2610	2.2610
			2277607.66	260968.56	100.00	100.00	25.5869	25.5869

Figure S13. Gas chromatographic report for the purity of N_2 used.

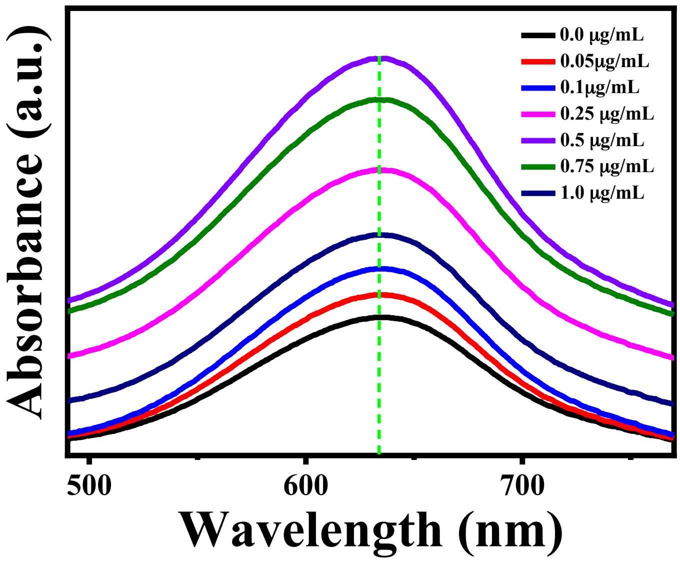

Figure S14. UV-vis absorption presenting the different known concentrations of NH_4^+ in 1 M Na_2SO_4 with indophenol blue indicator solutions after 1 h incubation under ambient conditions

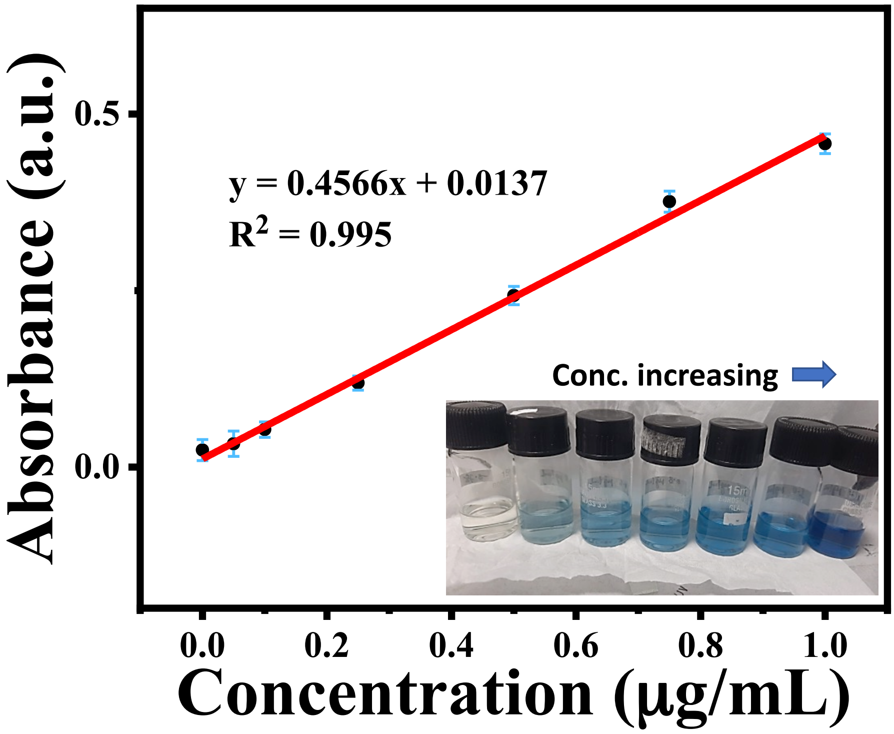

Figure S15. Calibration curve of ammonia – indophenol blue absorbance used in this study.

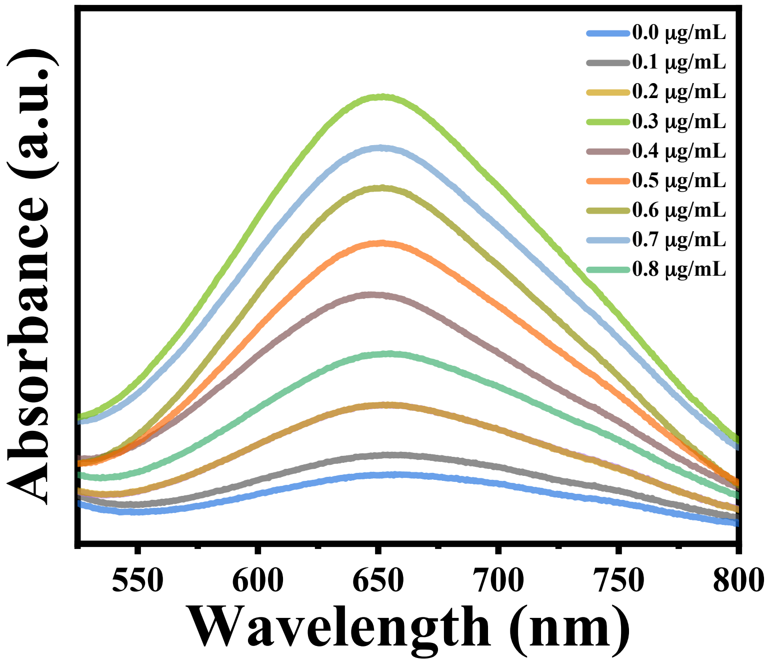

Figure S16. UV-vis absorption presenting the different known concentrations of NH_4^+ with indophenol-blue indicator in 0.05 M H_2SO_4 (acid trap) solutions after 1 h incubation under ambient conditions

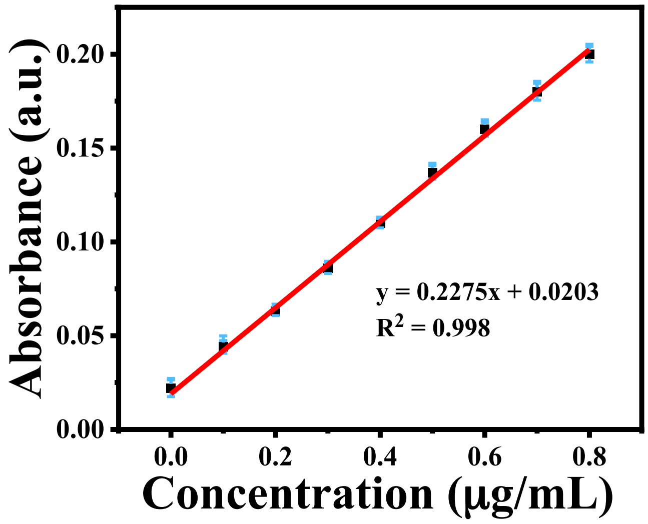

Figure S17. Calibration curve of ammonia – indophenol blue absorbance in (0.05 M H_2SO_4) acid trap solutions after 1 h incubation under ambient conditions

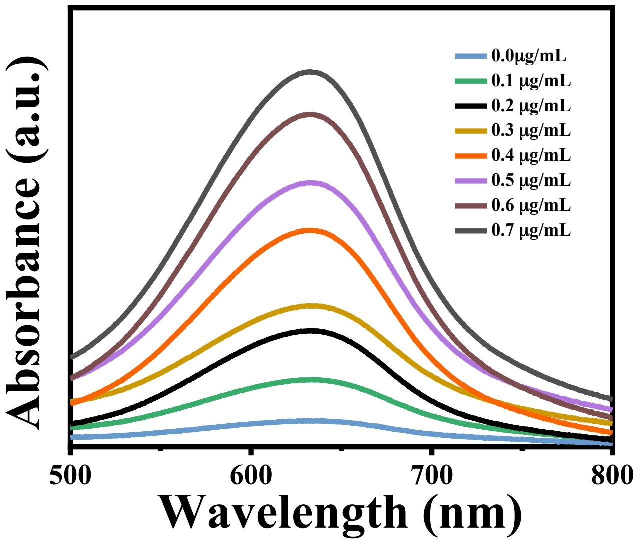

Figure S18. UV-vis absorption presenting the different known concentrations of NH_4^+ with indophenol-blue indicator in base trap solutions after 1 h incubation under ambient conditions.

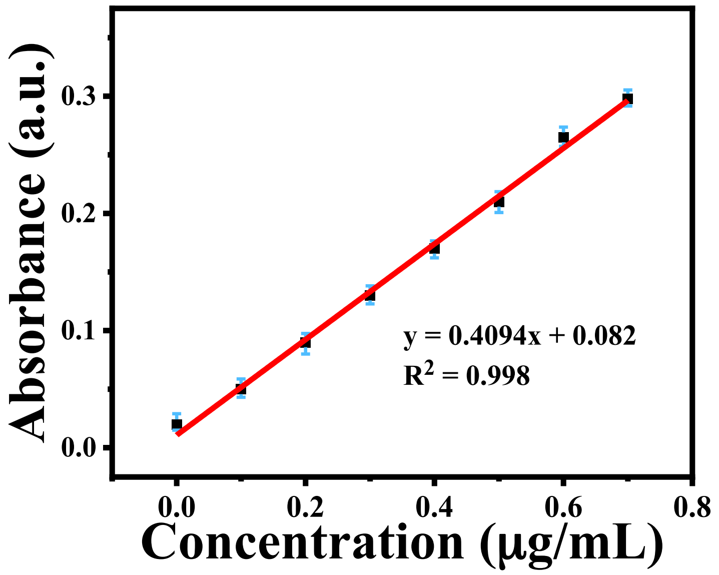

Figure S19. Calibration curve of ammonia – indophenol blue absorbance in base trap solutions after 1 h incubation under ambient conditions.

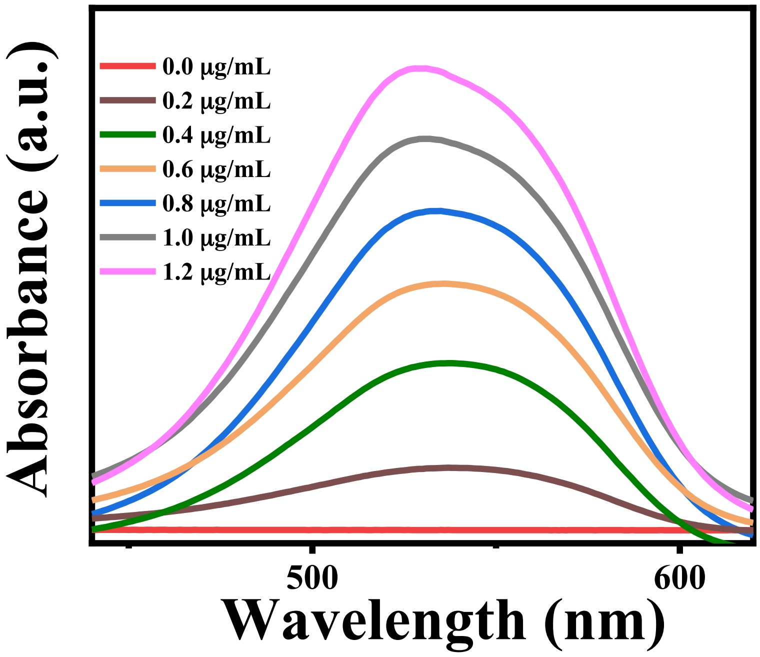

Figure S20. UV-vis absorption presenting the different known concentrations of NO_x with indophenol-blue indicator solutions after 1 h incubation under ambient conditions.

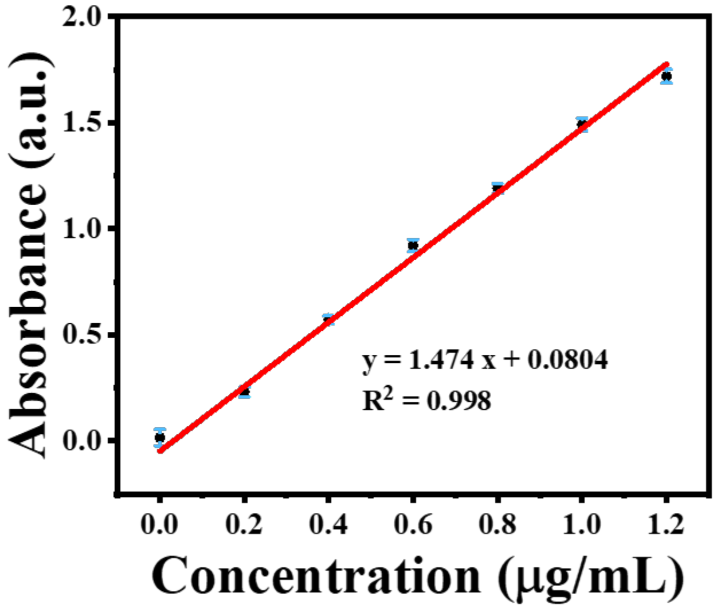

Figure S21. Calibration curve of ammonia – indophenol blue absorbance for solutions containing NO_x after 1 h incubation under ambient conditions.

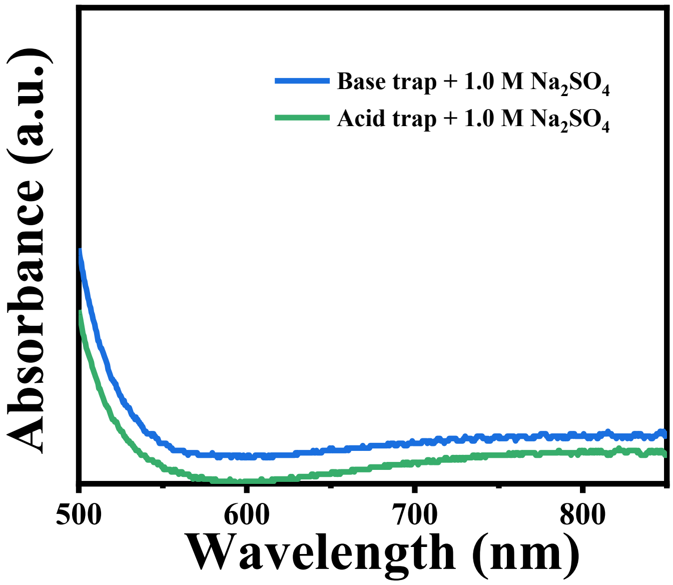

Figure S22. UV-vis absorption spectra of 0.1 M KOH (basic) and 0.05 M H_2SO_4 (acidic trap) + 1 M Na_2SO_4 of F-CuPc-G run at -0.2 V under ambient conditions.

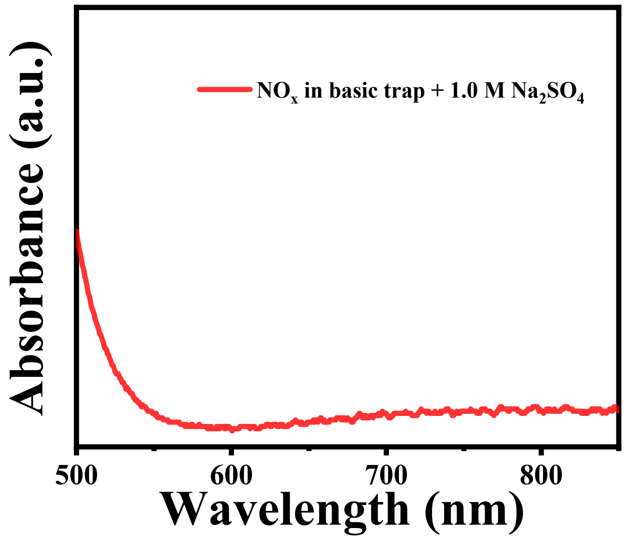

Figure S23. UV-vis absorption spectra of 0.1 KOH (basic trap) + 1 M Na_2SO_4 of F-CuPc-G run at -0.2 V under ambient conditions for detection of NO_x.

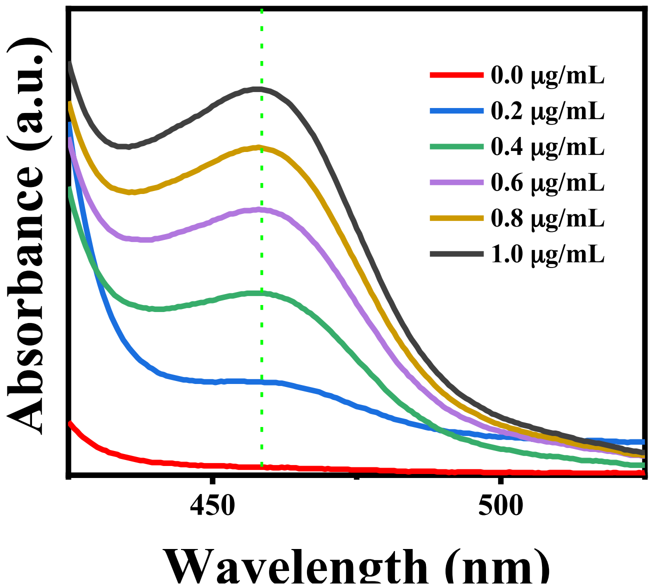

Figure S24. UV-vis absorption presenting the different known concentrations of N_2H_4 after 15 mins incubation under ambient conditions.

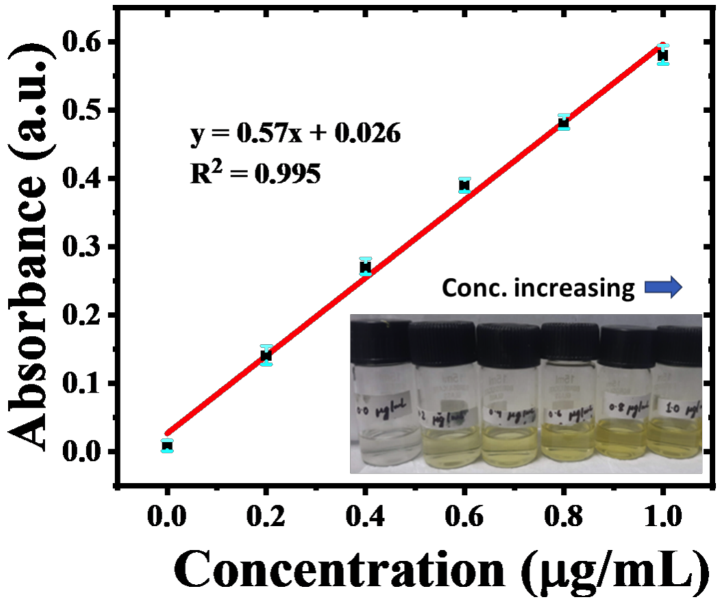

Figure S25. Calibration curve of hydrazine–absorbance used in this study.

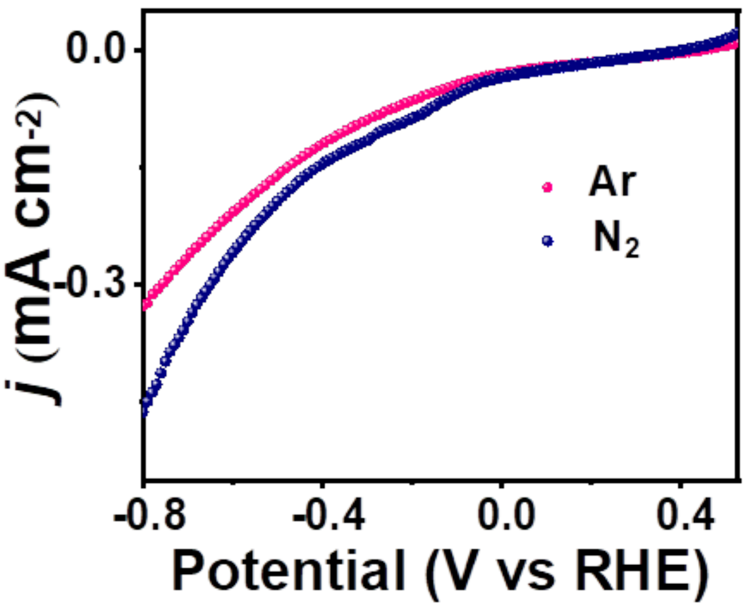

Figure S26. LSV response of F-CuPc-G in Ar and N_2 in 1 M Na_2SO_4.

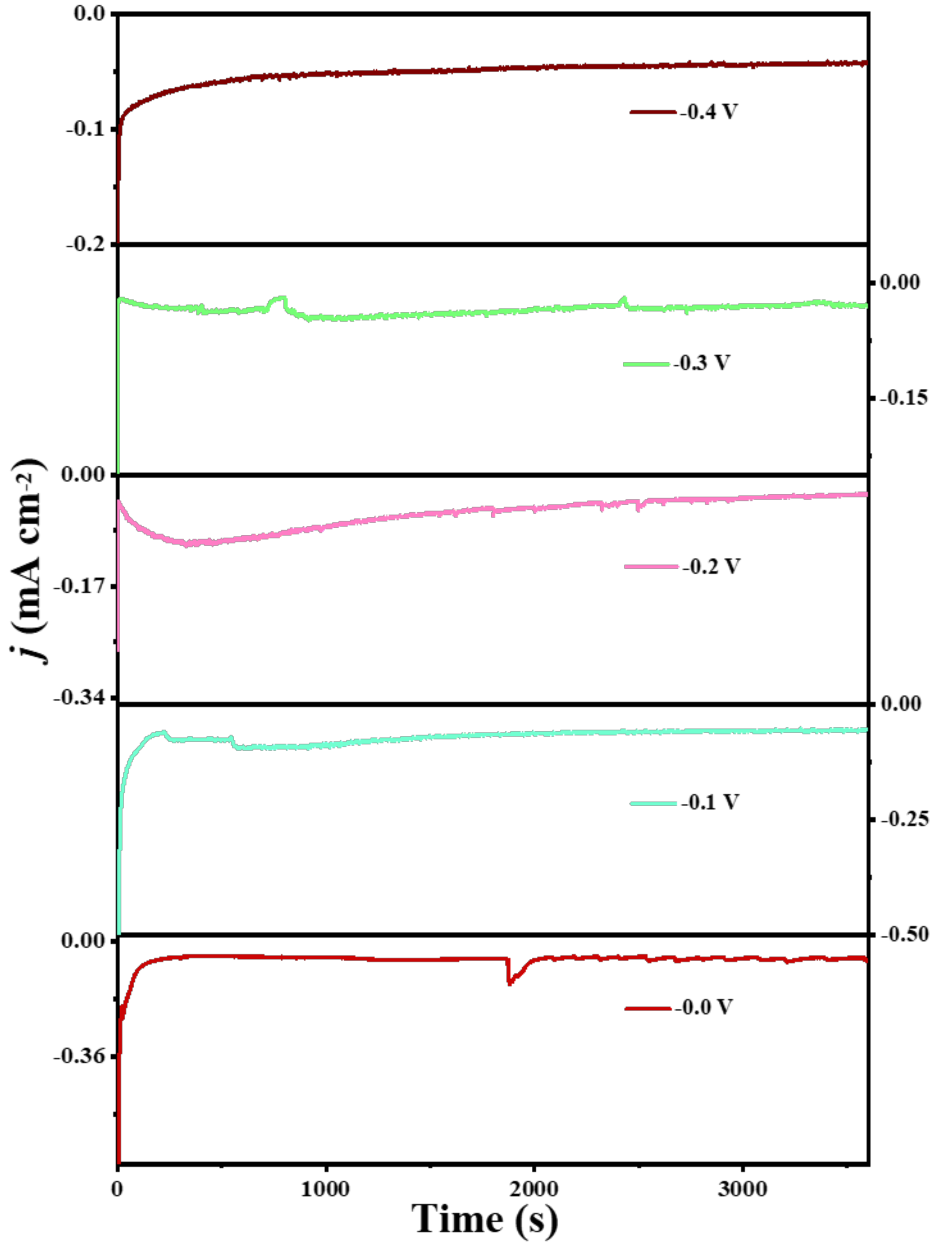

Figure S27. Chronoamperometric response of F-CuPc-G at different potentials in 1 M Na_2SO_4.

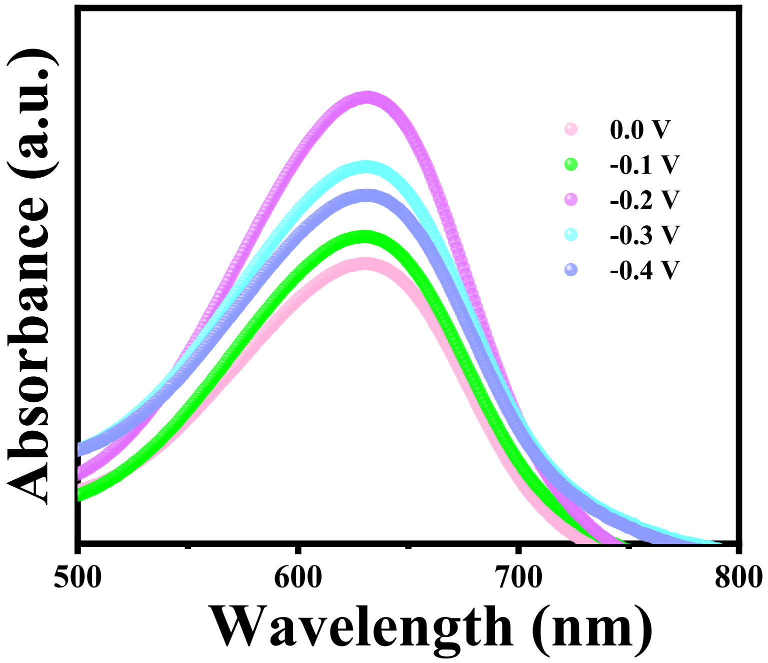

Figure S28. UV-vis absorption spectra of 1 M Na_2SO_4 containing different concentrations of NH_4^+ at different potentials with indophenol-blue indicator solutions after 1 hour chronoamperometric test.

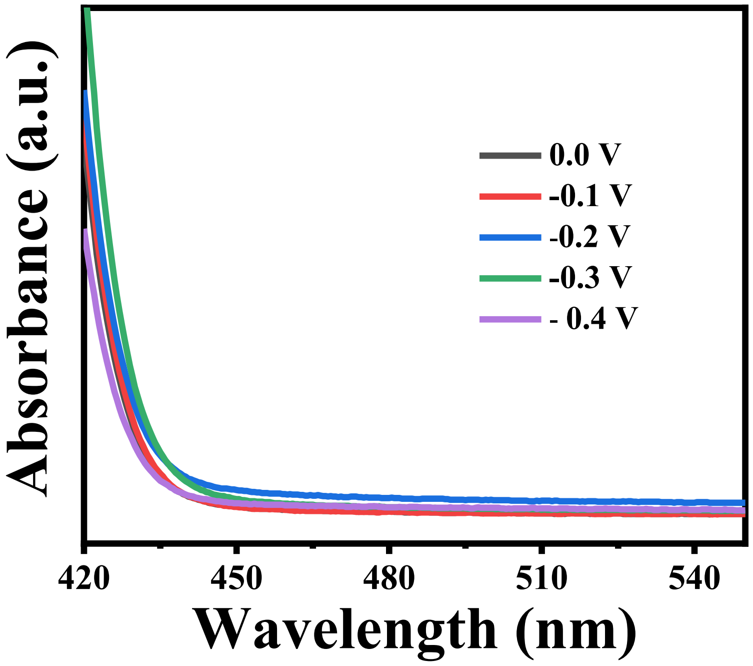

Figure S29. UV-vis absorption spectra of 1 M Na_2SO_4 containing different concentrations of N_2H_4 produced at different potentials after 15 mins of incubation under ambient conditions.

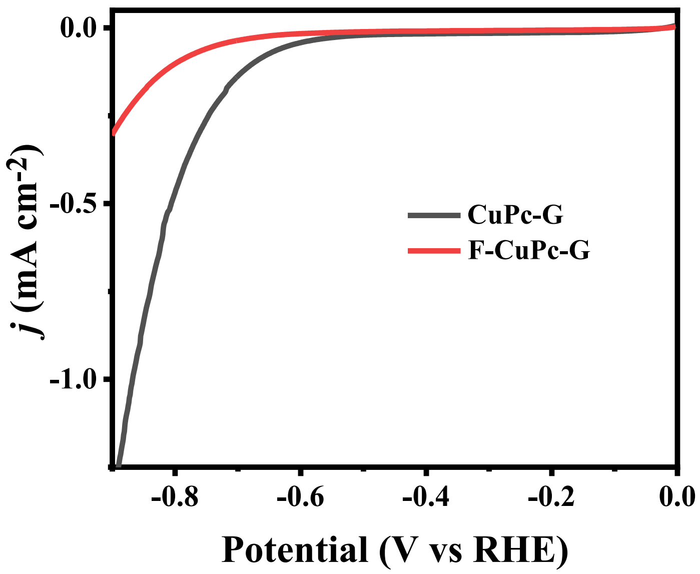

Figure S30. LSV of CuPc-G and F-CuPc-G taken at 5 mV s^{-1} in 1 M [illegible]

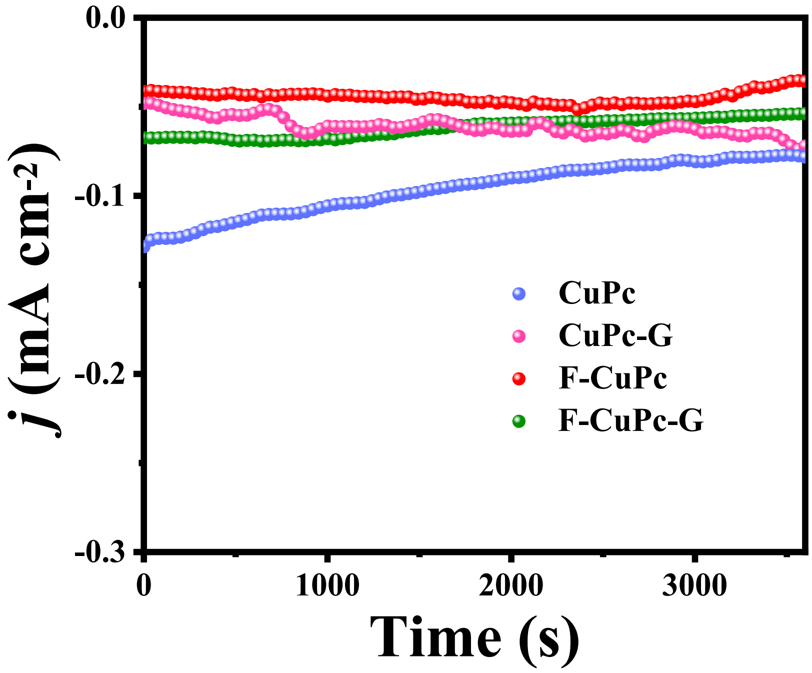

Figure S31. Chronoamperometric response of different control samples in 1 M Na_2SO_4.

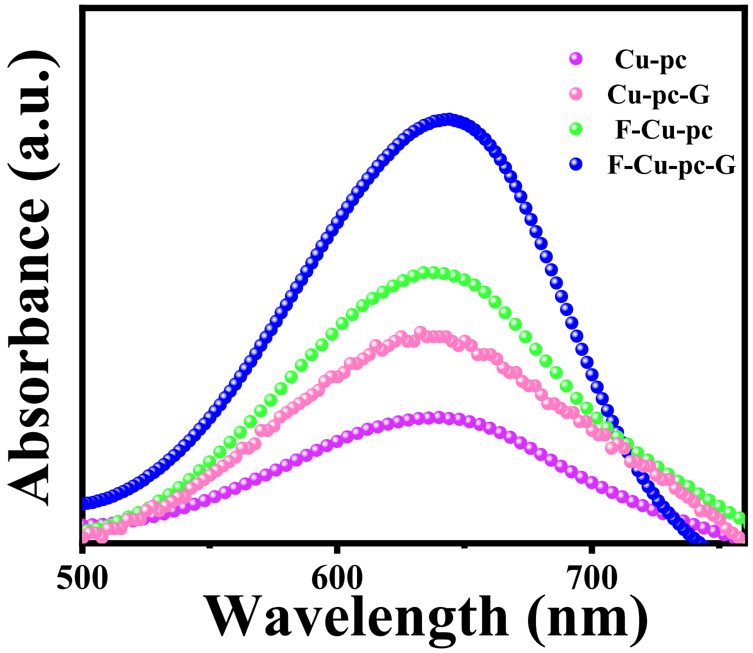

Figure S32. UV-vis absorption spectra of 1 M Na_2SO_4 containing different concentrations of NH_4^+ produced with different catalysts using indophenol-blue indicator solutions after 1 h incubation under ambient conditions.

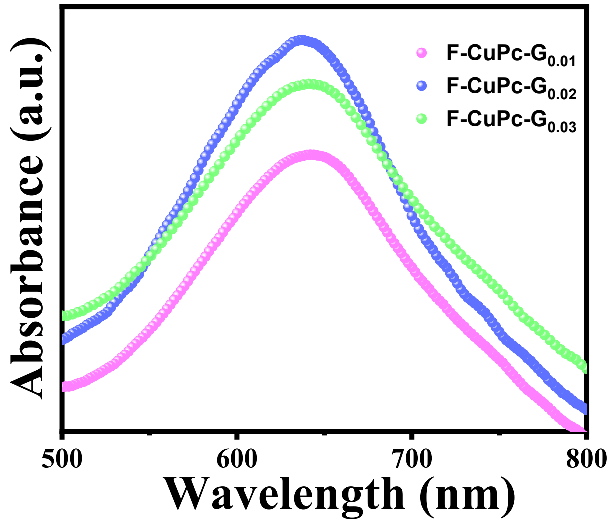

Figure S33. UV-vis absorption spectra of 1 M Na_2SO_4 containing different concentrations of NH_4^+ produced in control samples with different ratios of F-CuPc and graphene using indophenol-blue indicator solutions after 1 h incubation under ambient conditions.

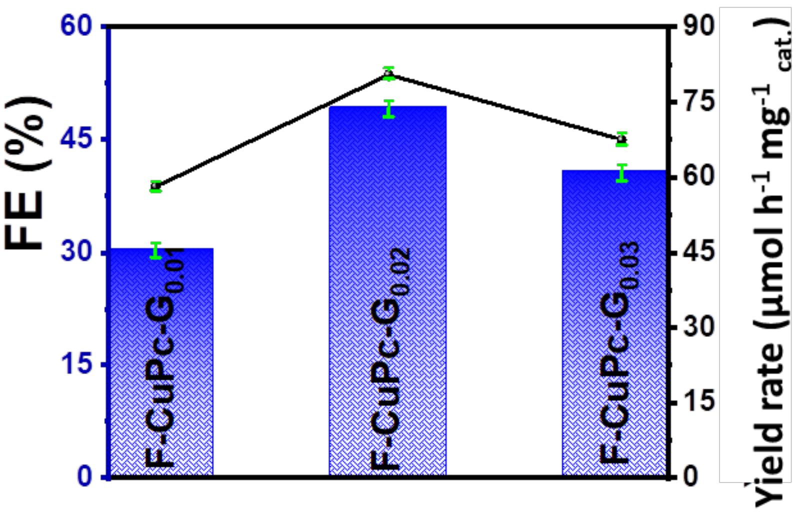

Figure S34. FE (%) and NH_3 yield rate of different control samples with different ratios of F-CuPc and graphene.

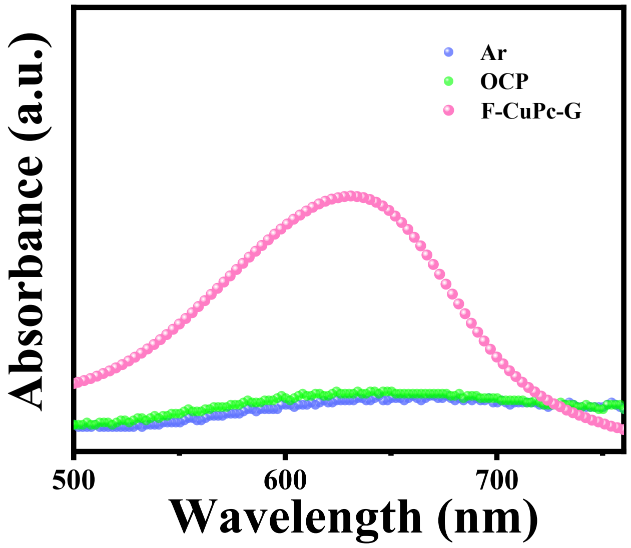

Figure S35. UV-vis absorption spectra of 1 M Na_2SO_4 containing different concentrations of NH_4^+ produced in argon and at OCP using indophenol-blue indicator solutions after 1 h incubation under ambient conditions.

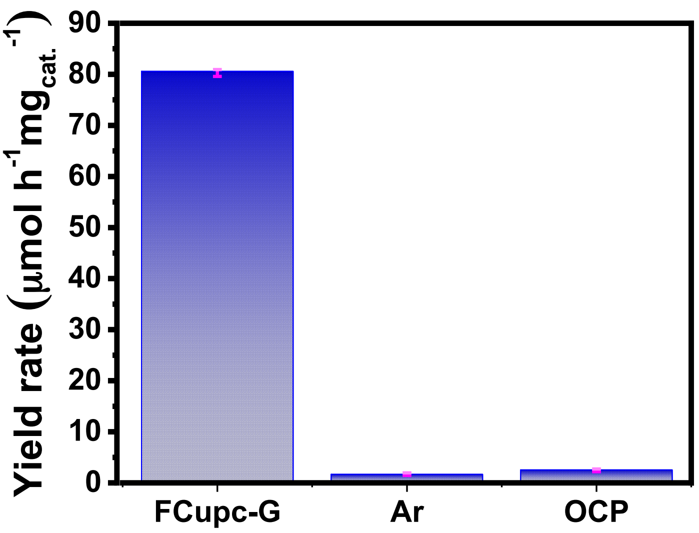

Figure S36. NH_3 yield rate of F-CuPc-G in N_2, Ar and at OCP.

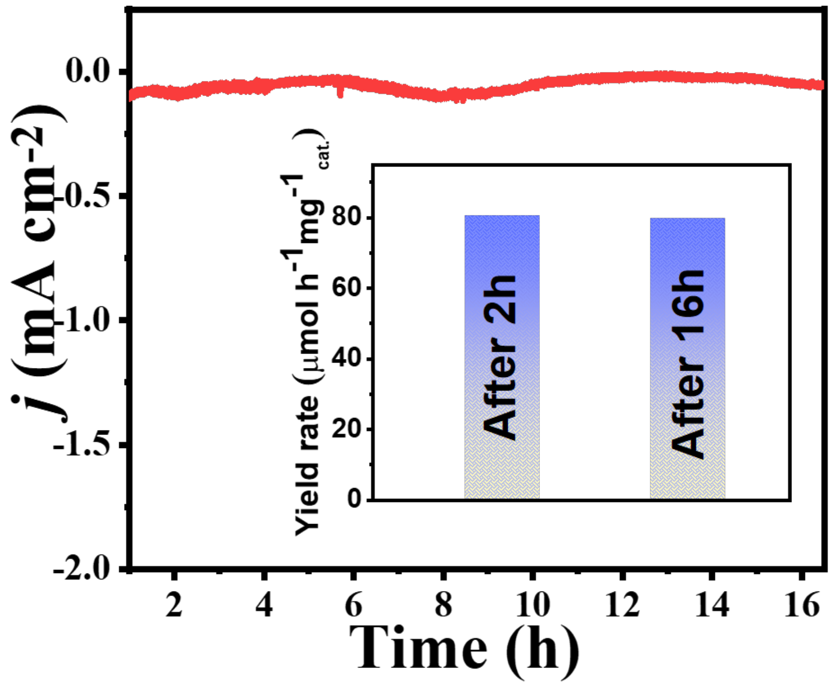

Figure S37. (a) Time-dependent current density curve for F-CuPc-G at –0.2 V for 16 h, inset: bar plot presenting the NH_3 yield rate for 1 h initially and after 16 h of NRR experiment.

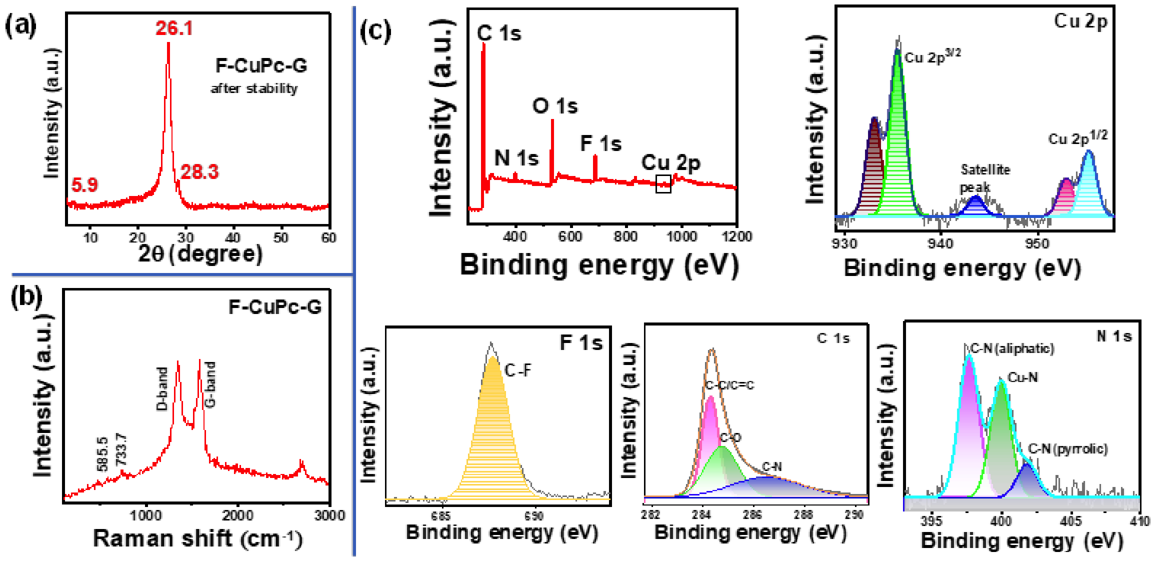

Figure S38. After stability (a) XRD pattern (b) Raman spectra of F-CuPc-G: (c) XPS spectra: full survey spectra of F-CuPc-G and high-resolution spectra of Cu 2p, F 1s, C 1s and N1s in F-CuPc-G.

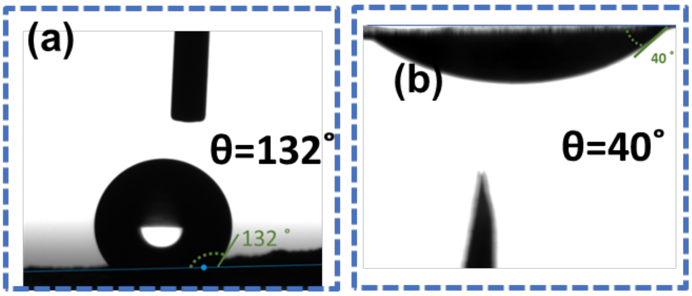

Figure S39. The contact angles of the (a) drop measured on the surface of F-CuPC-G; (b) N_2 bubble measured on the surface of F-CuPC-G.

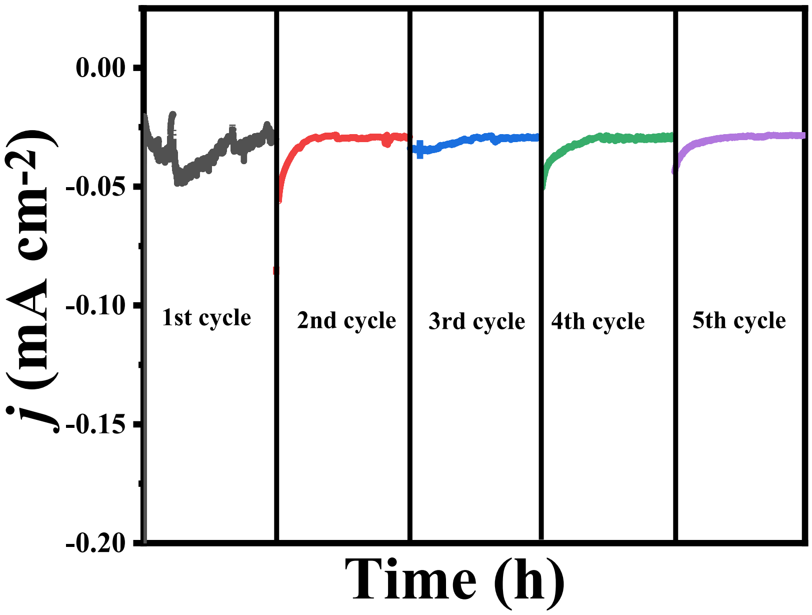

Figure S40. Chronoamperometric response of F-CuPc-G during five cycles of repeatability at -0.2 V potential in 1 M Na_2SO_4.

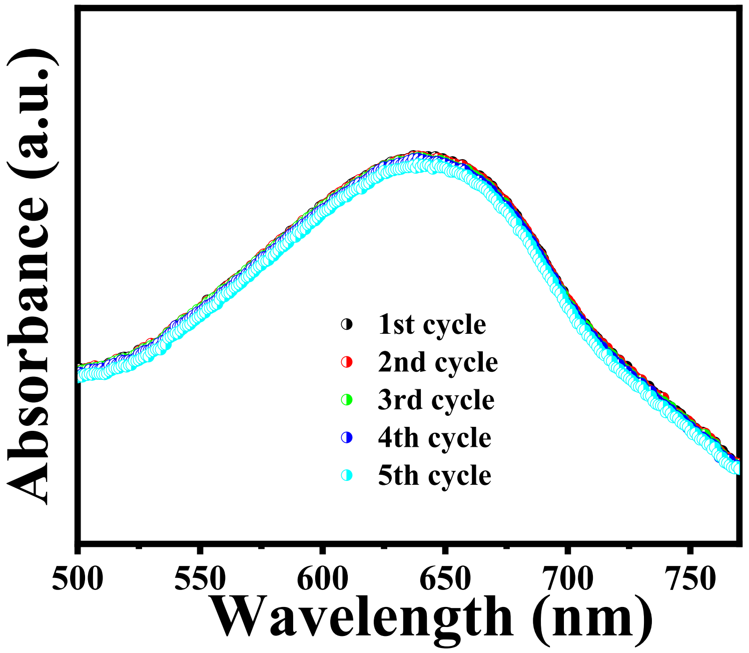

Figure S41. UV-vis absorption spectra of 1 M Na_2SO_4 containing NH_4^+ produced during different five cycles of repeatability at -0.2 V using indophenol-blue indicator solutions after 1 h incubation under ambient conditions

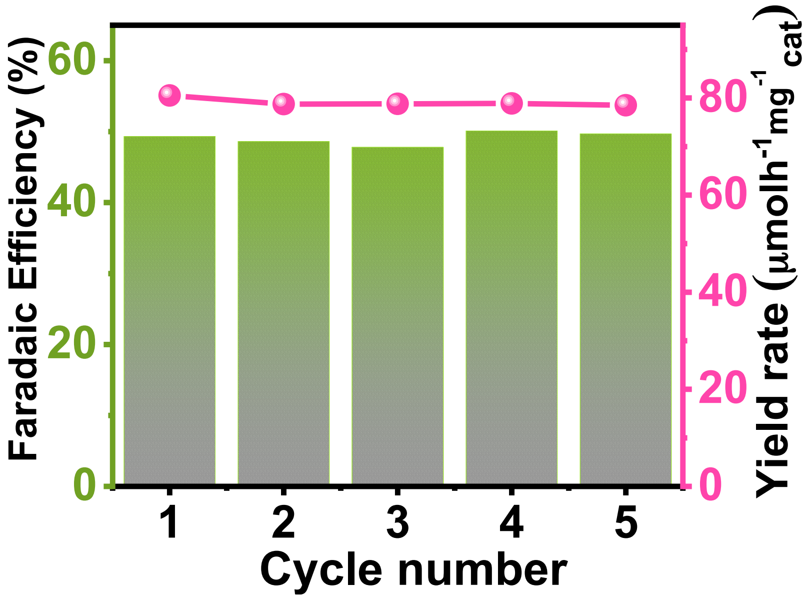

Figure S42. FE and NH_3 yield rate of five recycling tests for F-CuPc-G.

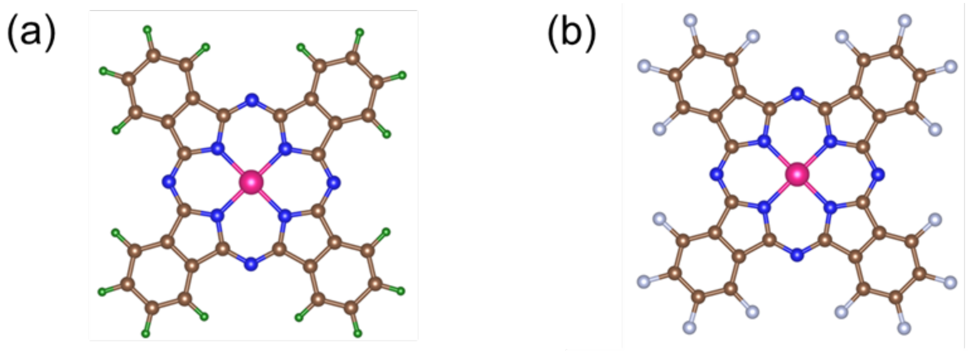

Figure S43. The Optimised model structures of (a) CuPc (b) F-CuPc.

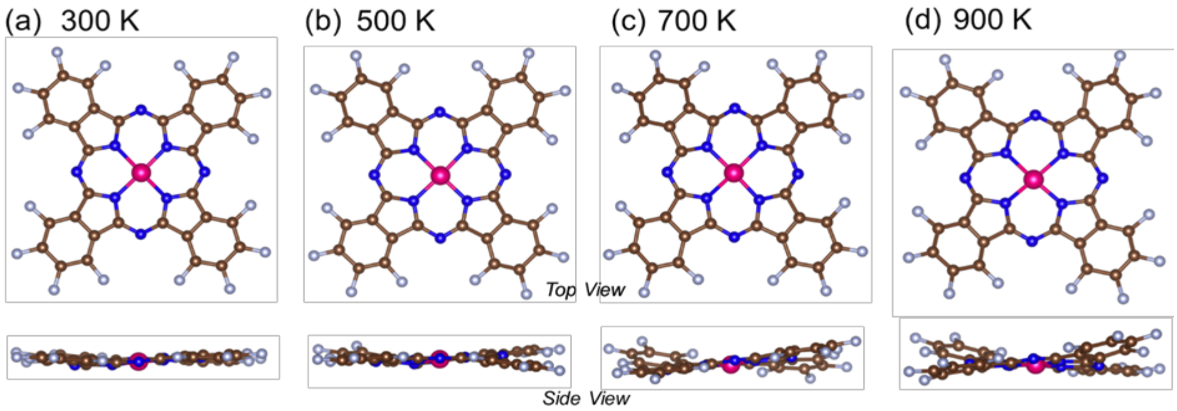

Figure S44. The optimized models of F-CuPc molecule at the temperature of (a) 300 K (b) 500 K (c) 700 K (d) 900 K in the molecular dynamic's simulation (e) Free Energy Profile of N_2 and NNH adsorption for CuPc and F-CuPc (f) Full NRR for F-CuPc.

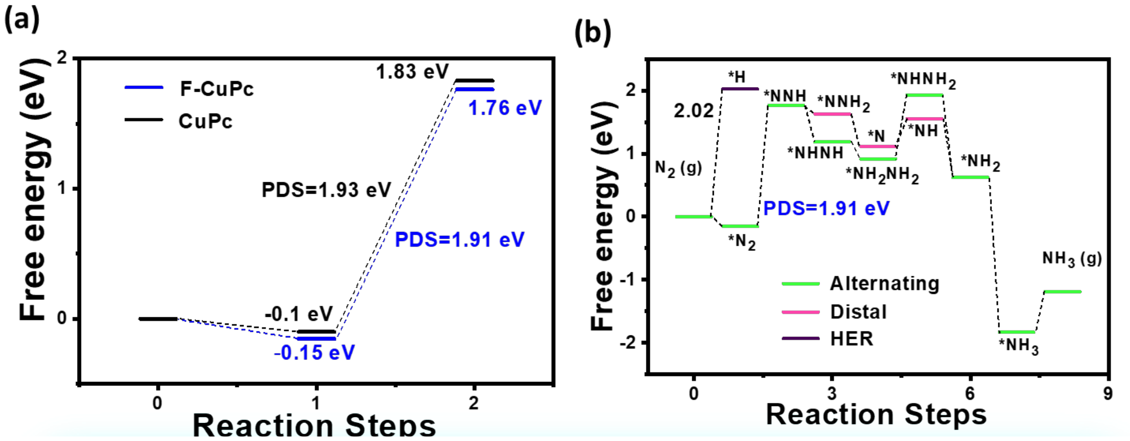

Figure S45. Free Energy Profile of N_2 and NNH adsorption for (a) CuPc and F-CuPc (b) Full NRR for F-CuPc.

Table S1. Cu-content present in the different exfoliated samples calculated by ICP-MS.

**Sample**	**Cu-content (in ppb) per 2mg of sample**	**Cu-content (in ppb) in 0.07 mg cat loaded on gc**
F-CuPc-$G_{0.01}$	3456	120.96
F-CuPc-$G_{0.02}$	4200	147
F-CuPc-$G_{0.03}$	5184	181.44

Table S2. Electrocatalytic NRR performance of F-CuPc-G at different potentials towards NRR in 1 M Na_2SO_4.

**Potential (RHE)**	**FE (%)**	**Yield rate (μmol h^{-1} mg^{-1} $_{cat}$)**
0.0 V	20.2	47.26
-0.1 V	24.6	49.3
-0.2 V	36.4	80.58
-0.3 V	49.3	60.18
-0.4 V	29.9	54.74

Table S3. Electrocatalytic NRR performance of different control samples towards NRR in 1 M Na_2SO_4.

**Sample**	**FE (%)**	**Yield rate (μmol h^{-1} mg^{-1}_{cat})**
F-Cu-pc-G	49.3	80.53
F-Cu-pc	36.9	52.5
Cu-pc	12.8	30
Cu-pc-G	28.9	45.1

Table S4. Literature comparison of various types of electrocatalysts used in NRR with F-CuPc-

Electrocatalyst	Electrolyte	FE (%)	Yield rate	Ref.
**Electrocatalysts having macrocycles**				
Ti-COF	0.05 M HCl	34.62%	26.89 μg h−1 mg−1cat.	5
CoPc NTs	0.1 M HCl	27.7%	107.9 μg h^{-1} mg^{-1}_{cat}	6
NiPc nanorods	0.1 M HCl	25 %	85 μg h^{-1} $mg^{-1}_{cat.}$	7
**Fluorinated electrocatalysts**				
F-doped carbon	0.05 M H_2SO_4	54.8%	197.7 μg h^{-1} $mg^{-1}_{cat.}$	8
d-FG	0.1 M Na_2SO_4	4.2%	9.3 μg h^{-1} $mg^{-1}_{cat.}$	9
F-SnO_2/C	0.1 M Na_2SO_4	8.6%	19.3 μg h^{-1} $mg^{-1}_{cat.}$	10
**HER suppressing electrocatalysts**				
Fe SACs on MoS_2	0.1 M KCl	31.6% ± 2%	97.5 ± 6 μg h^{-1} cm^{-2}	11
F-$Ti_3C_2T_x$ MXene	0.01 M Na_2SO_4	7.4%	2.81× 10^{-5} μmol·s^{-1} ·cm^{-2}	12
Ru/MoS_2	0.01 M HCl	17.6%	1.14 × 10^{-10} mol cm^{-2} s^{-1}	13
Zr^{4+}-doped anatase TiO_2	0.1 M KOH	17.3%	8.90 μg·h^{-1}·cm^{-2}	14
F-CuPc-G	1.0 M Na_2SO_4	67.15 %	78.75 μg h^{-1} $mg_{cat.}$ 1	This work

Table S5. Elemental composition of various elements present in F-CuPc-G obtained from XPS analysis.

Element	Atomic % (Before stability)	Atomic % (After stability)
C	78.7	78.44
N	4.28	4.22
O	9.28	9.74
F	7.42	7.27
Cu	0.32	0.34

References

1 G. Kresse and D. Joubert, *Phys. Rev. B*, 1999, **59**, 1758.

2 P. E. Blöchl, *Phys. Rev. B*, 1994, **50**, 17953–17979.

3 J. P. Perdew, K. Burke and M. Ernzerhof, *Phys. Rev. Lett.*, 1996, **77**, 3865–3868.

4 S. Kapse, S. Janwari, U. V Waghmare and R. Thapa, *Appl. Catal. B Environ.*, 2021, **286**, 119866.

5 M. Jiang, L. Han, P. Peng, Y. Hu, Y. Xiong, C. Mi, Z. Tie, Z. Xiang and Z. Jin, *Nano Lett.*, 2022, **22**, 372–379.

6 U. K. Ghorai, S. Paul, B. Ghorai, A. Adalder, S. Kapse, R. Thapa, A. Nagendra and A. Gain, *ACS Nano*, 2021, **15**, 5230–5239.

7 S. Murmu, S. Paul, S. Kapse, R. Thapa, S. Chattopadhyay, A. N., S. N. Jha, D. Bhattacharyya and U. K. Ghorai, *J. Mater. Chem. A*, 2021, **9**, 14477–14484.

8 Y. Liu, Q. Li, X. Guo, X. Kong, J. Ke, M. Chi, Q. Li, Z. Geng, J. Zeng, Y. Liu, Q. Li, X. Guo, X. Kong, J. Ke, M. Chi, Z. Geng and J. Zeng, *Adv. Mater.*, 2020, **32**, 1907690.

9 J. Zhao, J. Yang, L. Ji, H. Wang, H. Chen, Z. Niu, Q. Liu, T. Li, G. Cui and X. Sun, *Chem. Commun.*, 2019, **55**, 4266–4269.

10 Y. P. Liu, Y. B. Li, H. Zhang and K. Chu, *Inorg. Chem.*, 2019, **58**, 10424–10431.

11 J. Li, S. Chen, F. Quan, G. Zhan, F. Jia, Z. Ai and L. Zhang, *Chem*, 2020, **6**, 885–901.

12 Y. Ding, J. Zhang, A. Guan, Q. Wang, S. Li, A. M. Al-Enizi, L. Qian, L. Zhang and G. Zheng, *Nano Converg.*, 2021, **8**, 1–7.

13 B. H. R. Suryanto, D. Wang, L. M. Azofra, M. Harb, L. Cavallo, R. Jalili, D. R. G. Mitchell, M. Chatti and D. R. MacFarlane, *ACS Energy Lett.*, 2019, **4**, 430–435.

14 N. Cao, Z. Chen, K. Zang, J. Xu, J. Zhong, J. Luo, X. Xu and G. Zheng, *Nat. Commun. 2019 101*, 2019, **10**, 1–12.
